# A new approach for atmospheric turbulence removal using low-rank matrix factorization

**DOI:** 10.7717/peerj-cs.1713

**Published:** 2024-01-31

**Authors:** Mahdi Jafaei, Amirhassan Monadjemi, Payman Moallem, Mohammad Saeed Ehsani

**Affiliations:** 1 Department of Artificial Intelligence, Faculty of Computer Engineering, University of Isfahan, Isfahan, Iran; 2 School of Continuing and Lifelong Education, National University of Singapore, Kent Ridge, Singapore; 3 Department of Electrical Engineering, Faculty of Engineering, University of Isfahan, Isfahan, Iran

**Keywords:** Atmospheric turbulence, Distortion, Image restoration, Mixture of Gaussian, Spatiotemporal varying blur, Transformation matrix

## Abstract

In this article, a novel method for removing atmospheric turbulence from a sequence of turbulent images and restoring a high-quality image is presented. Turbulence is modeled using two factors: the geometric transformation of pixel locations represents the distortion, and the varying pixel brightness represents spatiotemporal varying blur. The main framework of the proposed method involves the utilization of low-rank matrix factorization, which achieves the modeling of both the geometric transformation of pixels and the spatiotemporal varying blur through an iterative process. In the proposed method, the initial step involves the selection of a subset of images using the random sample consensus method. Subsequently, estimation of the mixture of Gaussian noise parameters takes place. Following this, a window is chosen around each pixel based on the entropy of the surrounding region. Within this window, the transformation matrix is locally estimated. Lastly, by considering both the noise and the estimated geometric transformations of the selected images, an estimation of a low-rank matrix is conducted. This estimation process leads to the production of a turbulence-free image. The experimental results were obtained from both real and simulated datasets. These results demonstrated the efficacy of the proposed method in mitigating substantial geometrical distortions. Furthermore, the method showcased the ability to improve spatiotemporal varying blur and effectively restore the details present in the original image.

## Introduction

**Figure 1:**
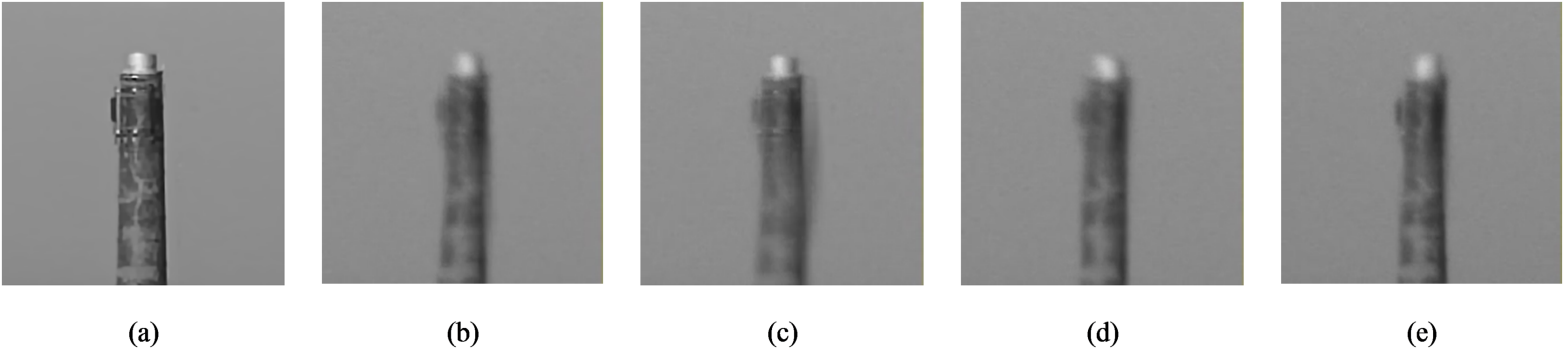
Demonstration of turbulent images caused by atmospheric interventions. (A) Turbulence-free image. (B–E) Some examples of turbulent images caused by atmospheric interventions. Photo credit: Chiman Kwan.

Atmospheric turbulence arises as a result of temperature variations within the air. This phenomenon can be observed on hot summer days with the movement of hot air masses from an asphalt road. Moving hot air masses between cold air masses in an environment gives rise to turbulence, evident in images captured under such weather conditions. This turbulence is manifested by the simultaneous occurrence of geometric distortion and blurring. The main underlying cause of this phenomenon is the variation of the refractive index of light within the air under varying conditions. The density of air, as the main cause of the refractive index, changes depending on the amount of moisture in the air and its temperature. Warmer and humid air has a lower density, resulting in a lower refractive index compared to colder and drier air (Pernechele, 2005). Hence, the turbulence of images increases with increasing thermal energy; however, aerosols such as steam, fog, dust, and so on can also exert a substantial impact on image quality. Figure 1 shows an example of a turbulence-free image and some examples of turbulent images captured from the identical scene.

Since the quality of an image can greatly affect the final results of any image processing algorithm, eliminating turbulence from images greases the wheels for higher accuracy. Turbulence removal can be used in many applications, such as long-distance surveillance applications concerning astronomy, the military, processing images transmitted from drones, surveillance, and medical imaging.

Astronomical images captured from large optical telescopes frequently encounter corruption due to the fluctuations in the refractive index within Earth’s atmosphere. For the purpose of image analysis, one of the initial pre-processing stages involves the removal of turbulence (Zhang, Zhao & Wang, 2011). In military applications, the utilization of long-range images renders them susceptible to turbulence. These images can be aerial images that are acquired from drones or land-to-land images. Consequently, the implementation of a turbulence removal process becomes imperative (Arora & Singh, 2018).

In surveillance and security systems, adverse weather conditions such as rain, fog, smoke, and haze can significantly undermine the clarity and efficacy of captured images. Consequently, these systems necessitate the integration of a turbulence removal mechanism to increase their accuracy (Arora & Singh, 2018). In the realm of medical imaging technologies, such as computer tomography (CT), nuclear magnetic resonance (NMR), and positron emission tomography (PET), the technique of image fusion is employed to generate a unified image from multiple viewpoints.

During the fusion process, the occurrence of distortion and blurring results in a decline in image quality. As a consequence, the implementation of turbulence removal can help medical experts to make safe decisions (Arora & Singh, 2018). The previous methods for removing turbulence from a set of images can be divided into three main categories:

In the first category of these methods, individual processing is performed on each image within the turbulent image set, with the application of the deblurring process. The second category employs a two-stage approach involving image selection (lucky frame and lucky region) and subsequent image composition. In these methods, the selection is based on criteria such as sharpness, and then images are combined using techniques like kernel regression and PCA. Ultimately, within the third category, the initial step involves generating a reference image from the input images. Subsequently, each input image is registered with this reference image. At this juncture, the registration process can involve either all the input images or a subset of pre-selected images. The registered images are merged to yield a singular composite image. Subsequently, the ultimate image is generated by subjecting the image from the preceding step to a deconvolution process. The subsequent list showcases some of the cutting-edge methods introduced within this domain.

Li, Mersereau & Simske (2007) employed the PCA method to conduct deconvolution on multi-channel input images. This approach overlooks phase data and filters out high frequencies, potentially leading to the restoration a few local textures. Aubailly et al. (2009) introduced a localized variation of the lucky frame technique, termed the lucky region. In this method, an image quality map is initially generated for each turbulent image. These maps are constructed based on a local sharpness metric. Subsequently, in each stage, the image quality map is compared with the fused image to select the optimal local quality (lucky region). Ultimately, this selected region is merged with the corresponding region of the fused image up to this stage. A primary challenge associated with this method lies in its substantial reliance on the lighting conditions of the images. Additionally, determining the composition parameters proves to be a complex endeavor.

Lau, Lai & Lui (2019a) devised a method for swiftly generating a high-quality image, devoid of the need for image registration. They achieved this through the utilization of a variational model and an appropriate energy function. This energy function incorporates a specific term designed to quantify the level of disparity between the extracted and subsampled images.

Oreifej et al. (2011) presented a two-step approach wherein the initial step involves computing the temporal average of the image sequence through an iterative procedure. In the subsequent step, the approach focuses on minimizing sparse errors, thereby effectively reducing noise, accomplished through rank minimization.

Mao & Gilles (2012) employed optical flow to estimate distortion and employed a non-local total variational model to mitigate blurring. Zhu & Milanfar (2012) produced a reference image by averaging a sequence of images. The non-rigid image registration was then applied individually to each image. The sequence of registered images was partitioned into overlapping patches with the local sharpness of each patch being evaluated based on its brightness variance. Subsequently, they employed temporal kernel regression to determine the ultimate selection of image patches. Ultimately, the blind deconvolution algorithm was implemented to alleviate image blur. This method had its limitations, including the reliance on the average sequence of images during the registration phase, which could potentially impact accuracy. Furthermore, it lacked the capability to rectify any registration errors that might emerge in the subsequent stages of processing.

Meinhardt-Llopis & Micheli (2014) initially extracted the modified parts of each pair of images using optical flow, and each part was wrapped with the mean vector of the corresponding part, yielding a new central image for every individual image. Ultimately, the resultant average image was employed as the reference image.

Xu et al. (2019) used low-rank matrix factorization (LRMF) (Meng & De La Torre, 2013) for detecting a moving object in a turbulent environmental setting. Their approach involved background subtraction from the images through LRMF. Following this, employing a pipeline method (Wang, Inigo & McVey, 1990), they proceeded to identify the moving object within the residual foreground.

Lau, Lai & Lui (2019b) made a sharp reference image from a sequence of images by formulation of a pertinent energy function. Additionally, they generated a new image sequence from the same set of images by aligning regions that were nearly sharp and exhibited minimal distortion.

Subsequently, employing this novel image sequence alongside the reference image, they applied the quasiconformal map and robust principal component analysis (RPCA) to the image registration procedure. Following successful image registration, they proceeded with the task of deblurring the lower rank regions of the images, accomplished through blind deconvolution. Ultimately, through the amalgamation of these processed images, the final image was generated. Xie et al. (2016) initially generated a high-quality reference image characterized by high sharpness and minimal noise. They achieved this through the utilization of low-rank decomposition. Subsequently, they engaged in a recursive optimization process for refining the reference image, utilizing a variational model. This model encompassed both local and non-local regularization elements, which significantly aided the image registration process. In the subsequent step, they used spatial-temporal kernel regression to produce a new image characterized by minimal blur. Ultimately, in the concluding step, the final output was generated using space-invariant blind deconvolution. Lou et al. (2013) employed a combined approach involving Sobolev gradient and Laplacian methods to remove turbulence. The Sobolev gradient was employed to eliminate blurriness from the images, while the Laplacian method rectified distortions. Essentially, they curbed oscillations while enhancing image sharpness. Subsequently, they applied the lucky frame method to yield the high-quality image. Zhang et al. (2018) introduced a novel turbulence removal framework termed complex steerable pyramid (CSP). Initially, they decomposed the incoming turbulent images through CSP, and subsequently computed an averaged reference image from the decomposed phase images. Employing the disparity between the phase of the current image and that of the reference image, they endeavored to minimize distortions. Deblurring was carried out through incorporation of local energy and displacement information, alongside CSP reconstruction that merged phase and amplitude information. Eventually, the final image was obtained using blind deconvolution.

Mao, Chimitt & Chan (2020) remove turbulence effects in three steps. Initially, they employ a non-local space-time averaging technique to construct a reference frame that retains moving objects. Subsequently, they generate a lucky frame using sharpness and consistency metrics. The sharpness metric identifies the sharpest patches, while the consistency metric eliminates residual jittering pixels stemming from the prior stage. Due to the presence of space-time varying blur within input frames and blurring artifacts introduced by previous processes, the ultimate step entails the utilization of blind deconvolution. Chak, Lau & Lui (2021) proposed the Wasserstein Generative Adversarial Network (WGAN) for atmospheric turbulence removal. The use of the ${l_1}\;$ norm as the cost function in GAN contributes to the preservation of essential textures within the restored image. A data augmentation step was incorporated as preprocessing by them to counter data scarcity. Additionally, the employment of a subsampling process resulted in enhanced and more accurate results for their proposed method. Hua et al. (2020) introduced a three-step methodology. The initial stage encompassed subsampling and reference image generation processes. Within this stage, two distinct and independent segments were employed to evaluate the degree of sharpness and geometric distortion. Subsequently, the non-rigid image registration technique was harnessed in the second stage to reduce geometric distortions. This step encompassed registering the sequences of sharply selected images from the initial stage to the reference image, achieved through estimation of deformation vectors. In the concluding stage, they sharpened the final image using the blind deconvolution method.

In the proposed method, the turbulence is modeled as

(1) $${x_{jk}} = \; D{(H\left( f \right))_{jk}} + {\varepsilon _{jk}}$$

In this equation, *x* denotes a turbulent image, while, *k* and *j* represent the pixel’s position. *H* signifies the blurring operator, *D* corresponds to the geometric distortion operator, *ε* represents the sensor’s noise, and *f* stands for the original image.

This article introduces a novel approach for turbulence removal within a sequence of images.

This approach effectively removes severe distortions and yields an image with prominently restored details. Unlike prior methodologies reliant on reference images and image registration for turbulence removal, this article presents a new framework that uses the concept of low-rank matrix factorization to remove turbulence. To achieve this objective, the modeling of geometric distortion and spatiotemporal varying blur is undertaken using the transformation matrix and mixture of Gaussian noise, respectively, during the computation of the low-rank matrix. Consequently, the calculation of the low-rank matrix encompasses two integral components: the estimation of blur, executed through the expectation-maximization (EM) method (Dempster, Laird & Rubin, 1977), and the estimation of distortion, accomplished *via* transformation matrix estimation. Within this approach, the initial step involves the adoption of the random sample consensus (RANSAC) method (Fischler & Bolles, 1981) to select a subset of optimal turbulent images from the turbulent images. Following this, parameters related to spatiotemporal varying blur, which are modeled as MOG (mixture of Gaussian) noise, are estimated through the EM method. The distortion in this method is modeled as the geometric transformation per pixel. Subsequently, in order to determine this transformation, a region is selected around each pixel. The size of this region is adaptive and is computed according to the entropy of the respective region. Based on the region’s entropy. Computation of the transformation matrix for each pixel—owing to the fact that distortions on image pixels stem from a finite collection of transformations—the totality of these transformations is modeled as a mixture of Gaussian (MOG) distribution. Consequently, the mean value of each Gaussian model is adopted as the corresponding transformation for that specific pixel. Then, the transformations are applied to each pixel, and through the utilization of the low-rank matrix factorization method, the collection of resulting images is decomposed. The ultimate image sets are acquired by multiplying the two components yielded from the decomposition process. Subsequently, through averaging the final image sets, the turbulence-free image is restored. Experimental results underline the effectiveness of the proposed method in reducing distortion and spatiotemporal varying blur. So, in a nutshell, the key contributions of the proposed method can be succinctly outlined as follows:

- Modeling turbulence through low-rank matrix factorization as the composition of geometrical distortion and spatiotemporal varying blur.
- Employing RANSAC for the selection of images exhibiting analogous turbulence models.

The remainder of this article is structured as follows: The initial section provides a detailed description of the proposed method. Subsequently, the subsequent section delves into the presentation of experiments, their outcomes, and comparative analyses with alternative turbulence removal algorithms. The ultimate section provides the conclusion. Also, a list of all the variables and symbols utilized within this article is presented in Table S1.

## Proposed method

In this article, the challenge of turbulence removal is modeled as a low-rank matrix factorization problem, in which the distortion and spatiotemporal varying blur are respectively modeled as a per-pixel transformation operator denoted as *τ* and a mixture of Gaussian noise. By employing this model, the turbulence-free image can be restored through the dual processes of noise elimination and the estimation of geometric transformations for each pixel. This section expounds upon the comprehensive procedure of the proposed method.

The initial subsection encompasses the discussion of the low-rank matrix factorization (LRMF) technique, which constitutes the crux of the proposed method. This technique is employed to decomposed input image sets, employing both expectation-maximization (EM) and weighted alternating least squares (WALS) methods for the estimation of parameters. Subsequently, the distortion model is referred to, describing how to obtain the transformation matrix of each pixel. The following subsection explains how to use the RANSAC method to select the most suitable set of images from the input images. Ultimately, the overall process of the proposed method is expounded upon in comprehensive detail.

### LRMF

Consider $X = \left[ {{x_1}, \ldots ,{x_n}} \right] \in {R^{d \times n}}\;$ representing a sequence of turbulent images. Here, *n* stands for the number of images and *d* signifies the dimension of each turbulent image, with each image expressed as a vector. The input dataset can be decomposed into $X = U{V^T}$, where *U* is the basis matrix and *V* is the coefficient matrix (Yong et al., 2017). Consequently, each element within the input matrix *X* can be modeled as follows:

(2) $${x_{ij}} = \; {u_i}\; v_j^T + {\varepsilon _{ij}}$$

where ${u_i}$ and $\; {v_j}$ represent the ${i^{th}}\; {\rm and}\; {j^{th}}$ row vectors, respectively, of matrices *U* and *V*. The term ${\varepsilon _{ij}}\;$ corresponds to the noise within the ${j^{th}}$ pixel of the ${i^{th}}$ turbulent image. This noise is the spatiotemporal varying blur inherent in turbulence images that is modeled as a Mixture of Gaussians (MOG).

Hence, the noise can be formulated as follows:

(3) $${\varepsilon _{ij} \; \sim\;} \; \mathop \sum \nolimits_{k = 1}^K {\pi _k}\; N\; \left( {0,\; \sigma _k^2} \right)$$

where $N\; \left( {0,\; \sigma _k^2} \right)\;$ denotes a Gaussian distribution with zero mean and ${\sigma ^2}$ variance and ${\pi _k}\;$ signifies the mixing proportion of the MOG. By merging Eqs. (2) and (3), the following relationship is established:

(4) $${x_{ij}}\sim \sum \nolimits_{k = 1}^K {\pi _k}N({x_{ij}}|{u_i}v_j^T,\sigma _k^2)$$

Subsequently, by considering (Eq. (5)) as a cost function and finding its maximum value, the estimations for the Gaussian noise parameters (*Π*, *Σ*) and the low-rank matrices *U* and *V* are determined. To find the maximum value of Eq. (5), the EM method is employed, as elaborated upon in the subsequent section.

(5) $$\mathop {max } \limits_{U,V,\Pi ,\; \Sigma } L\left( {U,V,\Pi ,\; \Sigma } \right) = max\; \mathop \sum \nolimits_{i,j} \log \mathop \sum \nolimits_{k = 1}^K {\pi _k}\; \; N({x_{ij}}|{u_i}v_j^T,\sigma _k^2)$$

where $\Pi = \left\{ {{\pi _1},{\pi _2}, \ldots ,{\pi _K}} \right\},\;$ and $\Sigma = \left\{ {{\sigma _1},{\sigma _2}, \ldots ,{\sigma _K}} \right\}$.

### Estimation of LRMF model parameters using the EM method

Equation (5) can be solved through the utilization of the EM method. This iterative technique computes the parameters of Eq. (5) in two steps: the Expectation (E) step and the Maximization (M) step.

**E step:** Let ${z_{ijk}} \in \left\{ {0,1} \right\}$ $\left( {\mathop \sum \nolimits_k {z_{ijk}} = 1} \right)$: ${z_{ijk}}$ takes the value of one ( ${z_{ijk}} = 1$) if the ${\varepsilon _{ij}}\;$noise is generated by the ${k^{th}}$ Gaussian distribution. If not, it equals zero ( ${z_{ijk}} = 0$). So, the probability of the partial membership of the noise of data ${x_{ij}}\;$( ${\varepsilon _{ij}}$) to the ${k^{th}}$ Gaussian model can be formulated as follows:

(6) $$E\left( {{z_{ijk}}} \right) = {\gamma _{ijk}} = \displaystyle{{{\pi _k}N({x_{ij}}|{u_i}v_j^T,\sigma _k^2)} \over {\mathop \sum \nolimits_{k = 1}^K {\pi _k}N({x_{ij}}|{u_i}v_j^T,\sigma _k^2)}}$$

**M step 1 (updating *Π*, *Σ*):** To estimate the MOG parameters, including *Π* and *Σ*, Eq. (5) is reformulated as follows:

(7) $$p(X,Z|U,V,\Pi ,\Sigma ) = \mathop \prod \nolimits_{i,j} \mathop \sum \nolimits_{k = 1}^K {\left[ {{\pi _k}N({x_{ij}}|{u_i}v_j^T,\sigma _k^2)} \right]^{{z_{ijk}}}}$$

Recall that ${z_{ijk}}\;$ is equal to one if ${x_{ij}}\;$ is generated by the ${k^{th}}$ Gaussian model; otherwise, it is zero.

Now, as shown in Eqs. (8) and (9), through maximizing the logarithm of Eq. (7), the parameters *Π* and *Σ* can be estimated, as presented in Eq. (10).

(8) $$L\left( {X,Z{\rm |}U,V,\Pi ,\Sigma } \right) = \mathop \sum \limits_{i,j} \mathop \sum \nolimits_{k = 1}^K {z_{ijk}}(log {\pi _k}\; - log \sqrt {2\pi } {\sigma _k} - \; \displaystyle{{{{({x_{ij}} - {u_i}v_j^T)}^2}} \over {2\sigma _k^2}})$$

${E_z}L\left( {X,Z{\rm |}U,V,\Pi ,\Sigma } \right) = \mathop \sum \limits_{i,j} \mathop \sum \nolimits_{k = 1}^K E({z_{ijk}})(log {\pi _k} - log \sqrt {2\pi } {\sigma _k} - \; \displaystyle{{{{({x_{ij}} - {u_i}v_j^T)}^2}} \over {2\sigma _k^2}}$

(9) $$\hskip88pt  = \mathop \sum \limits_{i,j} \mathop \sum \nolimits_{k = 1}^K {\gamma _{ijk}} \left(log {\pi _k} - log \sqrt {2\pi } {\sigma _k} - \; \displaystyle{{{{({x_{ij}} - {u_i}v_j^T)}^2}} \over {2\sigma _k^2}}\right)$$

(10) $${\pi _k} = \displaystyle{{{N_k}} \over {\mathop \sum \nolimits_{k = 1}^K {N_k}}},\; \; \sigma _k^2 = \displaystyle{1 \over {{N_k}}}\mathop \sum \nolimits_{i,j} {\gamma _{ijk}}{\left( {{x_{ij}} - {u_i}v_j^T} \right)^2}$$

where ${N_k} = \mathop \sum \nolimits_{i,j} {\gamma _{ijk}}$.

**M step 2 (updating *U*, *V*):** Employing the acquired estimations for the parameters *Π* and *Σ*, the values of *U* and *V* are determined through the employment of the Weighted Alternating Least Squares (WALS) method (Pan et al., 2008), which is described in the subsequent subsection.

Hence, the general procedure for estimating the low-rank matrices *U* and *V* involves the initial random initialization of the *Π*, *Σ*, and *V* matrices. Subsequently, by employing the EM steps and updating the *U* and *V* matrices using Eqs. (15) and (16) respectively, the process continues until the convergence criterion is satisfied (Pan et al., 2008).

### Weighted alternating least squares (WALS)

Consider $X \!=\! \left[ {{x_1},{x_2}, \ldots ,{x_n}} \right] \in {R^{d \times n}}\;$ as the data matrix, and $W \!=\! \left[ {{w_1},{w_2}, \ldots ,{w_n}} \right]  \in {R^{d \times n}}\;$ as the matrix of non-negative weights. These weights are computed based on the estimated noise values for each pixel, and they are formulated as $W = \sqrt {\mathop \sum \nolimits_{k = 1}^K \displaystyle{{{\gamma _{ijk}}} \over {2\pi \sigma _k^2}}}$. A higher weight ${w_{ij}}$ indicates that the data ${x_{ij}}\;$ has a more influence on the generation of the restored image.

As demonstrated in the aforementioned equation, a greater value of ${\gamma _{ijk}}\;$ implies an increased likelihood of partial membership of the data ${x_{ij}}\;$ within the ${k^{th}}$ Gaussian model. Consequently, the weight assigned to this data also rises. The weighted low-rank matrix approximation of matrix *X* is computed by minimizing the subsequent function:

(11) $$L\left( Y \right) = \vert\vert W \odot \left( {X - Y} \right)\vert\vert_F^2$$

where $\vert\vert . \vert\vert_F^2$ signifies the Frobenius norm and $\odot$ represents the Hadamard product. Let’s assume that the matrix *Y*, comprising turbulence-free images, can be decomposed into two lower-rank *U* and *V* matrices ( $r \ll min\left( {n,d} \right)$): $Y = U{V^T}$. Consequently, Eq. (11) can be reformulated as follows:

(12) $$L\left( {U,V} \right) = \mathop \sum \nolimits_{ij} \left\vert\left\vert {w_{ij}} \odot \left( {{X_{ij}} - {U_i}V_j^T} \right) \right\vert\right\vert _F^2$$

To avoid overfitting, by adding new terms, Eq. (12) becomes the following:

(13) $$L\left( {U,V} \right) = \mathop \sum \nolimits_{ij} \left\vert\left\vert{w_{ij}} \odot \left( {{X_{ij}} - {U_i}V_j^T} \right)\right\vert\right\vert_F^2 + \lambda \left( \left\vert\left\vert {w_{ij}} \odot {U_i}\right\vert\right\vert_F^2\; + \left\vert\left\vert {w_{ij}} \odot {V_j}\right\vert\right\vert_F^2 \right)$$

Here, *λ* represent regularization parameter. By assuming *V* as a constant and solving the equation $\displaystyle{1 \over 2}\displaystyle{{\partial L\left( {U,V} \right)} \over {\partial {U_i}}} = 0$, the estimation of *U* is accomplished, as illustrated in Eq. (15):

(14) $$\displaystyle{1 \over 2}\displaystyle{{\partial L\left( {U,V} \right)} \over {\partial {U_i}}} = \; {U_i}\left( {{V^T}diag\left( {{w_i}} \right)V + \lambda \left( {\mathop \sum \nolimits_j {w_{ij}}} \right)I} \right) - {x_i}diag\left( {{w_i}} \right)V$$

(15) $${u_i} = {x_i}diag\left( {{w_i}} \right)V{\left( {{V^T}diag\left( {{w_i}} \right)V + \lambda \left( {\mathop \sum \nolimits_j {w_{ij}}} \right)I} \right)^{ - 1}},\; \; 1 \le i \le d$$

Here $diag\left( {{w_i}} \right) \in \; {R^{n \times n}}\;$ denotes a diagonal matrix with elements ${w_i}\;$ and *I* represents an identity matrix with dimensions $r \times r$.

By performing the same procedure, the value of *V* can also be calculated as follows:

(16) $${v_j} = x_j^Tdiag\left( {{w_j}} \right)U{\left( {{U^T}diag\left( {{w_j}} \right)U + \lambda \left( {\mathop \sum \nolimits_i {w_{ij}}} \right)I} \right)^{ - 1}},1 \le j \le n$$

Algorithm 1 outlines the pseudo-code for parameter estimation using the LRMF method.

**Algorithm 1:** MOG algorithm for LRMF.

*input*: $X=(x_1,x_2,\ldots x_n)\in R^{d\times n}$
*Randomly initialize* ${ \Pi },{ \Sigma },V$
*repeat*
$\left( {E\; Step} \right){:}\; \; \; Evaluate\; {\gamma _{ijk}}\; for\; i = 1, \ldots .,n,\; j = 1, \ldots ,d,\; k = 1, \ldots ,Ma{x_k}\; by$ Eq. (6)
$\left( {M\; Step\; for\; { \Pi },{ \Sigma }} \right){:}\; \; Evaluate\; {\pi _k},\sigma _k^2\; for\; \; k = 1, \ldots ,Ma{x_k}\; by$ Eq. (10)
$\left( {M\; Step\; for\; {\rm U},{\rm V}} \right){:}\; \; Evaluate\; U,V\; by\; solving\; \mathop {\min }\limits_{U,V} {\parallel} W \odot \left( {X - U{V^T}} \right){\parallel} _F^2$
* through WALS where* $W = \sqrt {\mathop \sum \nolimits_{k = 1}^{Ma{x_k}} \displaystyle{{{\gamma _{ijk}}} \over {2\pi \sigma _k^2}}} \; for\; i = 1, \ldots .,d,\; j = 1, \ldots ,n$
$\left( {Automatic\; Ma{x_k}\; tuning} \right){:}\; \; If\; KL\left( {N\left( {{\mu _i},\sigma _i^2} \right),N\left( {{\mu _j},\sigma _j^2} \right)} \right) < \varepsilon \; for\; some$
$i,j,\; then\; combine\; {i^{th}},{j^{th}}\; gaussian\; component\; into\; a\; unique$
$gaussian\; by\; letting\; {\pi _i} = \; \; {\pi _i} + {\pi _j},\; \; \sigma _i^2 = \left( {{\pi _i}\sigma _i^2 + {\pi _j}\sigma _j^2} \right)/\left( {{\pi _i} + {\pi _j}} \right)$
${\mu _i} = \; \displaystyle{{{\pi _i}{\mu _i} + {\pi _j}{\mu _j}} \over {{\pi _i} + {\pi _j}}}$
$removing\; the\; {j^{th}}gaussian\; from\; {\Pi },{ \Sigma }.\; let\; Ma{x_k} = \; Ma{x_k} - 1$
*until meet the stop criteria*
*return U, V*

Since the count of Gaussian models has a significant impact on both the convergence speed and the accuracy of the algorithm, the determination of the count for these models was conducted in an adaptive manner.

As stated in Meng & De La Torre (2013), the process commences with the initialization of the count of Gaussian models, set at a value suitable for modeling the noise distribution (denoted as $Ma{x_k}$). Subsequent to each iteration of the EM algorithm, the distance between each pair of Gaussian distributions is computed employing the Kullback-Leibler (KL) divergence technique. If the distance between the two Gaussian models falls below the pre-established threshold, these models are regarded as similar, prompting their amalgamation. Given that the mean value of the Gaussian distributions utilized for noise modeling is consistently taken as zero, the amalgamation of Gaussian models necessitates the computation solely of the standard deviation and the mixing proportion for the resultant hybrid Gaussian model.

### Distortion model

Alongside inducing spatiotemporal varying blur, turbulence also introduces image distortion. Distortion encompasses abrupt shifts in the signal that lead to its degradation. Distortions in turbulent images emerge when the pixel displacement within the local vicinity can be modeled as a geometric transformation. Determining the optimal alignment for each pixel reduces distortion and strives to minimize the rank of the transformed image. With regard to the transformed image, the optimization equation for LRMF is presented as follows:

(17) $$\mathop {min } \limits_{U,V} \left\vert\left\vert W \odot {\left( {x \circ \tau - U{V^T}} \right)} \right\vert\right\vert_F $$

As $x \circ \tau$ involves a nonlinear operation, it is very difficult to optimize it within the same formulation. However, considering the slight changes in *τ*, by linearizing (Eq. (17)), the iterative estimation of *τ* becomes feasible, as delineated in Eq. (18).

(18) $$X \circ \left( {\tau + \Delta \tau } \right) \simeq X \circ \tau + \mathop \sum \nolimits_{i = 1}^n {J_i}\Delta {\tau _i}$$

where ${J_i}\;$ represents the Jacobian matrix corresponding to the ${i^{th}}$ image with respect to the transformation parameters $\; {\tau _i}$:

(19) $${{\rm J}_{\rm i}} = \displaystyle{\partial \over {\partial {\rm \varepsilon }}}({{\rm x}_{\rm i}} \circ {\rm \varepsilon }){{\rm / }_{{\rm \varepsilon } = {{\rm \tau }_{\rm i}}}}$$

The Robust Alignment by Sparse and Low-rank decomposition (RASL) method (Peng et al., 2012) has demonstrated the robustness of this approximation in cases of linearly correlated images, even in the presence of drastic variations in brightness and occlusion. Considering Eq. (19), the modification of the optimization function (Eq. (17)) for the ${i^{th}}$ image transpires as follows:

(20) $$\mathop {min }\limits_{U,V} \left\vert\left\vert {W_i} \odot {\left( {{x_i} \circ {\tau _i} + {J_i}\Delta {\tau _i} - UV_i^T} \right)}\right\vert\right\vert_F$$

where ${V_i}\; ,\; {\rm and}\; {W_i}$ denote the ${i^{th}}$ columns of matrices *V* and *W*, respectively, pertaining to the ${i^{th}}$ image ( ${x_i}$). With the known values of *U* and *V*, the cost function is established in Eq. (21) to estimate the transformation matrix:

(21) $$\mathop {min }\limits_{\Delta \tau } {\left\vert\left\vert e \right\vert\right\vert_1}, \; e = {x_i} \circ {\tau _i} + {J_i}\Delta {\tau _i} - UV_i^T$$

As Eq. (21) is a variation of the least absolute deviation problem, it can be effectively solved using the Alternating Direction Method of Multipliers (ADMM) method (Boyd, Parikh & Chu, 2011).

Subsequently, by formulating the Lagrangian function as Eq. (22) and incorporating the input values of $U,{V_i},$ ${{\rm \tau }_{\rm i}}\; ,\;$ and ${J_i}$, the optimal value of $\Delta {\tau ^{P + 1}}$ is computed utilizing the ADMM approach by Eq. (23):

(22) $$L\left( {U,V,e,\Delta \tau ,\lambda } \right) = \; {\left\vert\left\vert e \right\vert\right\vert_1} + {\lambda ^T}  h\left( {e,\Delta \tau } \right) + \displaystyle{\mu \over 2}  \left\vert\left\vert h\left( {e,\Delta \tau } \right)\right\vert\right\vert_2^2$$

where $h\left( {e,\Delta \tau } \right) = UV_i^T + e - {x_i} \circ {\tau _i} - {J_i}\Delta {\tau _i}$.

(23) $$\Delta {\tau ^{P + 1}} = {\left( {{J_i}J_i^T} \right)^{ - 1}}J_i^T\left( {UV_i^T + {e^P} - {x_i} \circ {\tau _i} + \displaystyle{1 \over {{\mu ^P}}}{\lambda ^P}} \right)$$

where $\; {e^{P + 1}} = {S_{\textstyle{1 \over {{\mu ^P}}}}}\left( {{x_i} \circ {\tau _i} + {J_i}\Delta \tau _i^{P + 1} - UV_i^T - \displaystyle{1 \over {{\mu ^P}}}{\lambda ^P}} \right)$, ${\lambda ^{P + 1}} = {\lambda ^P} + {\mu ^P}h\left( {{e^{P + 1}},\Delta \tau _i^{P + 1}} \right)$, and ${\mu ^{P + 1}} = \rho {\mu ^P}$.

In these equations, ${S_{\textstyle{1 \over {{\mu ^P}}}}}$ stands for the elementwise soft thresholding operator (as described in Boyd, Boyd & Vandenberghe (2004)). Here, $\; \rho > 1\;$ signifies the penalty value that increases the *μ* value in the ascending order, and *P* denotes the step within the ADMM algorithm. The overall procedure of this estimation is depicted in Algorithm 2.

**Algorithm 2:** ADMM for estimate $\Delta \tau$.

*input:* $U, V, x, \tau$
*initialization*: $e^1=0, \Delta \tau^1=0, \lambda^1=0, \mu=1$
*Cache* $Q = {\left( {{J^T}J} \right)^{ - 1}}{J^T}$
$for\; p = 1{:}a\; do$
$update\; \Delta \tau {:}\; \; \; \; \Delta {\tau ^{p + 1}} = Q\left( {UV_i^T + {e^T} - {x_i} \circ {\tau _i} + {1 \over \mu }{\lambda ^P}} \right)$
$update\; e{:}\; \; \; \; {e^{p + 1}} = {S_{{1 \over {{\mu ^P}}}}}\left( {{x_i} \circ {\tau _i} + {J_i}\Delta {\tau ^{p + 1}} - UV_i^T - \displaystyle{1 \over \mu }{\lambda ^P}} \right)$
$update\; \lambda {:}\; \; \; \; {\lambda ^{p + 1}} = {\lambda ^p} + \mu h\left( {{e^{p + 1}},\Delta {\tau ^{p + 1}}} \right)$
$update\; \mu {:}\; \; \; \; \mu = \rho \mu$
$if\; \vert\vert h{\left( {{e^{p + 1}},\Delta {\tau ^{p + 1}}} \right)\vert\vert_2} \le \; {\varepsilon ^{tollerance}}\; then$
* converge and break the loop*
* end if*
*end for*
$return\; \; \Delta {\tau ^*} = \Delta {\tau ^{p + 1}}$

As previously mentioned, the task entails determining the locally optimal transformation matrix for every pixel. Thus, for each image pixel, a 3 × 3 transformation matrix needs to be identified to effectively model the distortions. Rather than focusing on the transformation of individual pixels, it is feasible to model the transformation of a window encompassing the pixel at its center. After the application of the transformation matrix to each window, the value of each transformed pixel ( ${{\rm x}_{\rm i}} \circ {{\rm \tau }_{\rm i}}$) within the ${i^{th}}$ image corresponds to the median value across all windows containing that pixel.

To achieve this goal, the sliding windows approach is employed. The dimensions of each window is adaptive depending on the entropy of the window. Initially, the window’s size is set to the minimum size ${W_{min}}$ and it expands until its entropy becomes lower than $\alpha$ percent ( $0 < \alpha < 1$) of the image’s entropy. The maximum size allowed for windows is ${W_{max}}$. ${W_{min}},$ ${W_{max}}$ and $\alpha$ constitute the free parameters of the proposed method. Since higher entropy in the image indicates more intricate details, lower entropy (signifying image smoothness) results in greater uncertainty when calculating the transformation matrix. Thus, if the window’s size is expanded to the maximum permissible extent without surpassing α percent of the overall image entropy, the calculation of the transformation matrix for that window is skipped. Instead, its value is approximated using the transformation matrix from neighboring windows. Despite distortion implying abrupt shifts in pixel positions, these changes cannot be very drastic. Consequently, a mechanism must be considered for removing transformation matrices that yield substantial pixel displacements. To address this, the mean and variance of all estimated $\Delta {\tau _i}\;$ matrices are calculated. Those matrices exceeding a threshold of *β* percent, in terms of their distance from the mean value, relative to the total variance, are designated as outliers. Subsequently, pixels with the $\Delta {\tau _i}$ matrix that are identified as outliers (pixels with a window size equal to ${W_{max}}$ and those with a $\Delta {\tau _i}\;$ matrix with a drastic value) are assigned the mean value of $\Delta {\tau _i}$ matrices from neighboring pixels. Ultimately, the transformation matrix for each window is determined by accumulating the estimated $\Delta {\tau _i}$ values from different iterations. ( $\tau _i^{k + 1} = \Delta {\tau _i} + \tau _i^k$).

Given that the distortion affecting pixels arises from a limited range of distinct transformations, a MOG model is computed using the estimated transformation matrices. This MOG model serves as a representation of all the distortions affecting image pixels. Employing the computed MOG, the final transformation matrix for each window is established as the mean value of the Gaussian model, with the transformation matrix of that window being a member of said Gaussian model.

As a reminder, similar to the adaptive determination of the number of Gaussian models for noise (as outlined in Algorithm 1), the number of Gaussian models for distortions is also computed adaptively using the same approach. The comprehensive process of distortion removal is presented in Algorithm 3.

**Algorithm 3:** Removing distortion from image.

*input*: $x,U,V,\tau$
$extract\; subregion\; of\; image\; x\; using\; sliding\; windows\; based\; on\; the\; entropy\; of\; each $
$pixel\; as\; {f_j}\; ,j = 1, \ldots ,d$
$for\; each\; {f_j}{:}$
$if\; {f_j}\; satisfy\; entropy\; carcumestion\;$
* calculate* $\Delta\tau_j$ *using* Algorithm 2
* else*
* set* $\Delta\tau_j$ *as outlier*
* end if*
*end for*
*using variance and average of all* $\Delta\tau_j$ *to detect outlier ones*
*for each* $f_j$:
* if* $\Delta\tau_j$ *is outlier*
* calculate* $\Delta\tau_j$ *using* $\Delta\tau$ *matrix of neighboring windows*
* end if*
*end for*
$\tau_j= \tau_j+\Delta \tau_j$
*estimate MOG using* $\tau_j$ *and assign mean of MOG models to each windows*
*for each pixel index j in x*
* L = set of all j^th^ pixel exist in all transformed windows*
* j^th^* pixel *of* $x\circ \tau$ = median(L)
*end for*
*return $x\circ \tau$*

### Selecting a subset of images using the RANdom SAmple Consensus (RANSAC) method

Turbulent images can exhibit varying degrees of distortion and blurring due to different conditions and intensities. Consequently, identifying and excluding images that exhibit distinct turbulence patterns can lead to improved outcomes. The RANSAC method is employed within the proposed approach to achieve this objective (refer to Algorithm 4). To accomplish this, a subset of *m* images $\left( {m \ll n} \right)\;$) is randomly selected, and the proposed method (as described in Algorithm 5) is applied to this subset, yielding a restored image (denoted as *r1*). Subsequently, each of the remaining images is processed using Algorithm 5 along with the initial set of *m* images. This leads to the generation of an output matrix $R = U{V^T},\;$ containing $m + 1\;$ images. Instead of averaging the images, the ${\left( {m + 1} \right)^{th}}$ image is selected as the final image for the process.

**Algorithm 4:** RANSAC method to best subset image selection.

*input*: $X=[x_1,x_2,\ldots ,x_n]$
Initialization: r1 as restored image by averaging on m restored images, r2 as $(m+1)^{th}$ restored image
for *k* = 1:*C*
* select m^i^ images randomly*
* for* $l=1{:}n-m$
* calculate r*1: *apply* Algorithm 5 *on m^i^ images*
* calculate r*2: *apply* Algorithm 5 *on m^i^ images in addition to each remaining image* $\{g_j=1,\ldots,n-m\}$
* calculate C*1*_j_*: *calculate similarity of r*1 *and r*2 *images using cross correlation*
* calculate C*2*_j_*: *calculate noise distribution distance of m and m* + 1 *image sets using KL divergence*
* calculate C*3*_j_*: *calculate transformation distribution distance of m and m* + 1 *image sets using KL divergence*
* add g_i_ image to support set of m^i^ images if weighted average of* $C1_j,C2_j, C3_j$ *is less than predefined threshold*
* end for*
*end for*
*select* $\{m^i=1,\ldots , C\}$ that have largest support set as m* and related support set as s*
$return\, \{m^*+s^*\}$

**Algorithm 5:** Calculating low rank matrix using LRMF method.

*input:* $X=[x_1,x_2,\ldots ,x_n]$
$initilization:\; \; \; {x_i} \circ \tau _i^1 = {x_i}\; ,\; random\; initilization\; {V^1},{{ \Sigma }^1},{{ \Pi }^1}$
*repeat*:
* step* 1: *estimate U*, *V*
* use* Algorithm 1 *to estimate* ${U^{k + 1}},{V^{k + 1}},{{ \Sigma }^{k + 1}},{{ \Pi }^{k + 1}}$ *based on* ${U^k},{V^k},{{ \Sigma }^k},{{ \Pi }^k},\; {x_i} \circ \tau _i^k$
* step 2: estimate* $\tau$
* use* Algorithm 3 *to estimate* ${x_i} \circ \tau _i^{k + 1}$ $based\; on\; \; {U^{k + 1}},{V^{k + 1}}$
*until meet the stop criteria*
$return\; {R^*} = {U^{k + 1}}{V^{k + 1}}^T$

By utilizing cross-correlation, the similarity between the restored image (*r1*) and the ${\left( {m + 1} \right)^{th}}$ image is calculated, denoted as *C1*. Similarly, the distances between two noise distributions of the m images and the $m + 1\;$ images and two transformation matrix distributions $\left( \tau \right)\;$of the m images and the $m + 1$ images are computed using the Kullback-Leibler (KL) divergence. These are respectively termed *C2* and *C3*.

If the weighted average of these three criteria (*C1*, *C2*, and *C3*) falls below a predetermined threshold, this image is regarded as a member of the support set of the initial m images. The procedure for calculating the KL divergence between two MOG distributions is elaborated in Appendix A.

This process is iterated multiple times, and in the end, the *m* images with the largest support set are selected. The selected $m\;$ images and their associated support set are combined to create the new subset. The final restored image is obtained by applying the proposed method to this refined subset. In this process, the excluded images are the ones that have different turbulence characteristics compared to the others. It is important to note that the excluded images may not necessarily have lower turbulence than those that were not selected. Nonetheless, due to the fact that the selected images have the same conditions compared to each other, empirical evidence indicates that the proposed method efficiently estimates the blur and distortion characteristics within this subset, leading to an improved output.

### Overall procedure

As stated, the turbulence present in the images is modeled as being influenced by two distinct factors: distortion, characterized by geometric transformations that alter pixel positions, and spatiotemporal varying blur, which leads to variations in pixel brightness values.

**Figure 2:**
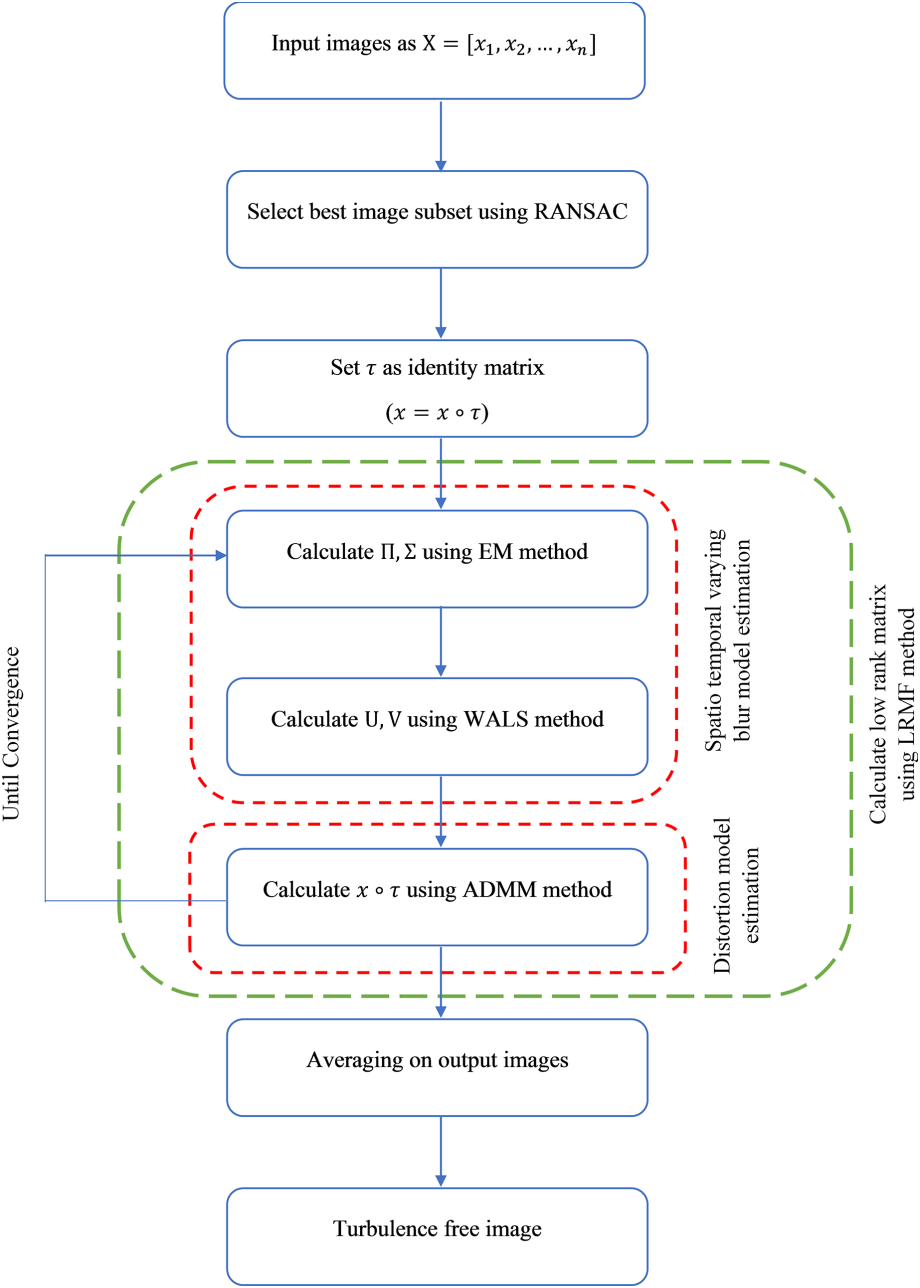
Overall procedure of proposed method.

Taking into account the methodologies discussed in the LRMF and distortion model sections, the comprehensive procedure of the proposed approach for removing turbulence in a collection of images captured from a single scene can be outlined as follows:

To begin, employ the RANSAC method as detailed in the selecting a subset of images using the RANSAC method section to identify the most suitable subset of input images. Then calculate the *R* matrix using optimization described by Eq. (17), which is the main optimization function of the proposed method, where $R = U{V^T}\;$ is the set of final images. Since the proposed procedure effectively eliminates turbulence, the *R* matrix includes *n* images by minimal turbulence. Consequently, a straightforward averaging of these *n* images yields the turbulence-free image (reference image). The estimation of both the *U* and *V* matrices entails an iterative methodology based on the process elucidated in the LRMF section. Nonetheless, due to the inherently nonlinear character of calculating the $x \circ \tau$ transformation, as expounded in the distortion model section, the transformation is executed iteratively by estimating ∆*τ* values. Consequently, to integrate these two iterative procedures, the previously outlined steps are executed concurrently. At the initiation of the procedure, all pixel transformation matrices are initially set as identity matrices ( $\forall i\; \; {\tau _i} = {\rm I}$). Subsequently, the algorithm commences with an iteration of Algorithm 1 (as detailed in the LRMF section) to estimate the values of ${V^k}\; {\rm and\; }\; {U^k}\;$ (with *k* representing the current iteration step of the algorithm). To achieve this, the initial step involves estimating the parameters of the MOG. Following this, the weights needed for the WALS method are calculated based on these parameters. Subsequently, utilizing the calculated ${V^k}\; {\rm and}\; {U^k}$ values obtained through the WALS method (as described in the LRMF section), the second step is executed. This involves computing *∆τ* for all images in accordance with Algorithm 2 (elaborated upon in the distortion model section), and subsequently generating ${x_i} \circ \tau _i^k$ (the transformed image in the ${k^{th}}$step). The algorithm’s detailed steps are presented in Algorithm 3. After this, the previously described procedure is repeated, and the *U* and *V* matrices are updated using the calculated ${x_i} \circ \tau _i^k$ values. This iterative process continues until the predetermined stopping condition is met. The complete procedure is visually depicted in Fig. 2. The main part of the proposed method is the calculation of the low rank matrix, which is shown by the green box in the mentioned figure. A detailed algorithmic representation of this part is provided in Algorithm 5.

## Experiments and results

In this section, we conduct experiments to evaluate the performance of the proposed method on both simulated and real datasets. The obtained results are then compared with the performance of several existing algorithms, namely Two-Stage (Oreifej et al., 2011), BNLTV (Mao & Gilles, 2012), NDL (Zhu & Milanfar, 2012), HVDKR (Xie et al., 2016), SGL (Lou et al., 2013), Centroid (Meinhardt-Llopis & Micheli, 2014), RPCA (Lau, Lai & Lui, 2019b), JSUB (Lau, Lai & Lui, 2019a), PCA-Based (Li, Mersereau & Simske, 2007), Lucky-Region (Aubailly et al., 2009), and UCSPF (Zhang et al., 2018). For this purpose, in this section, first, the datasets that were utilized for the experiments, are introduced. Subsequently, we evaluate the proposed method’s performance on these datasets and conduct a comparative analysis of the results with the aforementioned algorithms.

### Datasets

The evaluation of the proposed method encompasses both simulated and real datasets. The real datasets consist of the Chimney, Building, Moon-surface, and Water-tower sets, each of which includes corresponding ground truth images (Zhu & Milanfar, 2012). Meanwhile, the simulated datasets encompass the Car-front (Lau, Lai & Lui, 2019a) and Road (Lau, Lai & Lui, 2019a) sets. The simulated dataset is generated using two distinct approaches. The first method involves warping through a Gaussian deformation vector and introducing blurring through Gaussian noise. This dataset is referred to as the Gaussian simulated dataset. The second approach employs inter-modal and spatially correlated Zernike coefficients (Chimitt & Chan, 2020). This dataset is referred to as the Zernike simulated dataset. Further comprehensive information about these datasets is available in Appendix B.

### Criterion for comparison of algorithms

In this article, the two criteria of PSNR (peak signal-to-noise ratio) and SSIM (structural similarity index) are used to evaluate the proposed method. The PSNR criterion uses the root mean square error between two images. Larger PSNR values indicate greater similarity between images. Unlike PSNR, which uses error estimation, SSIM is a perception-based approach that incorporates important perceptual information such as structure and contrast.

The value of SSIM is in the range of [0, 1] and equals one if the two images are exactly equal.

### Free parameters and implementation details

The proposed method involves thirteen free parameters, which can be fine-tuned by experts and users who utilize the method. The specific values of these parameters, employed in all experiments within this section, are presented in Table 1. The free parameters can be classified into six distinct groups. The first group encompasses the convergence criteria of the EM method. This parameter involves a trade-off between runtime and algorithm accuracy. Given that turbulence removal is not a real-time computation but rather an offline problem, the chosen value strikes a balance between achieving satisfactory accuracy and ensuring a reasonable runtime. The intention is to avoid an excessively stringent threshold that could lead to impractical computation times while still maintaining attainable levels of accuracy. The second group of parameters pertains to the calculation of $\Delta \tau$ matrices and includes $\alpha$, ${W_{min}},$ and ${W_{max}},$, which are utilized in determining the size of the sliding window. Additionally, *β* is employed to identify outlier ∆*τ*. These parameter values were chosen through a process of trial and error, involving thorough examination of the datasets. It is important to note that the value of ${W_{min}}$ should be chosen small enough to encompass local transformation information, while ${W_{max}}$ should not be too large, as it might include a substantial portion of the image, causing the estimated transformation to become out of locality. The third group of free parameters is used to determine the optimal value of *∆τ* through the ADMM method. This group comprises parameters $\rho {\rm \; and\; }{\varepsilon ^{tolerance}}\;$ with their values set to the ones according to the recommendations of He et al. (2014). The fourth group of free parameters pertains to the number of MOG models used in noise and distortion estimation. This group encompasses the maximum number of models and the threshold for model integration, determined through a trial-and-error process. When selecting the value of the integration threshold, ensure that it is not too small, which would preserve similar models, and not excessively large, as it might lead to the merging of dissimilar models. The fifth group of free parameters encompasses the RANSAC parameters: the parameter *m* represents the minimum number of images required for the algorithm to function accurately. Through the examination of the datasets, it has been determined that the proposed method can effectively remove turbulence with a set of 25 images. Therefore, the parameter *m* has been set to 25. Within this group, a threshold value is defined for the purpose of identifying the support set. If the weighted mean of the three criteria introduced in selecting a subset of images using the RANSAC method section becomes lower than this threshold, the current image is regarded as a member of the support set of the initial set of *m* images. The value of this threshold is also determined through trial and error. The stopping criteria for the EM method (Algorithm 1), RANSAC method (Algorithm 4), and calculating low rank matrix (Algorithm 5) are the number of iterations, which are set to 40, 10, and 100 iterations, respectively. The sixth group of free parameters involves the λ parameter applied in the WALS method. The determination of this parameter value was achieved through an iterative process of trial and error, conducted specifically on the applied datasets.

**Table 1:** Free parameters of the proposed method and their values, which have been used in all experiments.

Value	Parameter name	Parameter group
$Ma{x_{Iter}} = 40$	Number of iterations	EM convergence criteria (Algorithm 4)
$\alpha = 0.56$	The ratio of entropy of each window w.r.t. the entropy of the whole image	Calculation of $\; \Delta {\rm \tau }$
${W_{min}} = {\rm max} \;\left(7,\displaystyle{1 \over {20}}\min \; \left( {width,height} \right)\right)$	Minimum size of sliding window	
${W_{max}} = {\rm max} \;\left(19,\displaystyle{1 \over 8}\min \; \left( {width,height} \right)\right)$	Maximum size of sliding window	
$\beta = 1.3$	Coefficient of selecting outlier transformation matrix	
$\rho = 2$	Penalty amount	Finding the optimal value of Δτ by the ADMM method
${\varepsilon ^{tollerance}} = {10^{ - 7}}$	Threshold of determination of convergence condition	
$\varepsilon = 0.15$	Merge threshold	Number of MOG models in estimating distortion and noise
$Ma{x_k} = 10$	Number of primary Gaussian models	
$m = 25$	Number of data choices	RANSAC method
$C = 10$	Number of iterations	
${\varepsilon ^{Ransac}} =$ 0.5	Similarity threshold for selecting support set	
λ = 0.0001	Regularization parameter	WALS method

### Compare the results of the proposed method with the existing methods

As mentioned, the results of the proposed method are compared with the results of the other eleven methods. The results of the Gaussian simulated dataset are compared with the Two-Stage, NDL, SGL, Centroid, RPCA, and JSUB methods, and the results of the Chimney and Building image sequences are compared with the PCA-Based, Lucky Region, Two-Stage, BNLTV, NDL, HVDKR, SGL, Centroid, and UCSPF methods. These comparative results are presented in Tables 2 and 3, respectively. The results of the proposed method on the sequences of the Moon-surface and Water-tower images are provided in Appendix C. As none of the previous methods reported results on these two sequences and their code was unavailable, only the results of the proposed method are presented for these datasets. Similarly, Table 4 contains results only for the Zernike simulated dataset. According to the results presented in Tables 2 and 3, it can be observed that the proposed method outperformed the previous methods in terms of the SSIM criterion for all datasets. However, in terms of the PSNR criterion, the proposed method exhibited lower performance compared to the previous methods for the Road and Chimney datasets.

**Table 2:** The results of the proposed method and other existing methods based on PSNR (first rows) and SSIM (second rows) criteria on Gaussian simulated datasets.

Sequence	Criteria	Two-stage	NDL	SGL	Centroid	RPCA	JSUB	Proposed
Car-front	PSNR	15.3815	19.9009	16.7093	19.5172	24.0959	20.9223	**25.1344**
	SSIM	0.5448	0.8136	0.6801	0.8163	0.9137	0.8375	**0.9460**
Road	PSNR	26.58	27.4061	23.9782	30.03	**33.8682**	32.1232	32.0174
	SSIM	0.7822	0.8036	0.7638	0.8608	0.9063	0.9005	**0.9226**
Mean	PSNR	20.9807	23.6535	20.3437	24.7736	**28.9820**	26.5227	28.5759
	SSIM	0.6635	0.8086	0.7219	0.8385	0.91	0.869	**0.9343**

**Note:**

The best result in each row is shown in bold.

**Table 3:** The results of the proposed method and other existing methods based on PSNR (first rows) and SSIM (second rows) criteria on the chimney and building sequences.

Sequence	Criteria	PCA-baced	Lucky region	Two-stage	BNLTV	NDL	HVDKR	SGL	Centroid	UCSPF	Proposed
Chimney	PSNR	16.2921	31.0421	30.6152	31.4314	31.0052	**32.0103**	28.4552	24.0133	31.0484	30.8352
	SSIM	0.0539	0.1137	0.1193	0.112	0.1075	0.1632	0.9065	0.8736	0.9155	**0.9231**
Building	PSNR	14.346	24.0679	25.0724	24.808	26.2145	26.4822	15.8501	24.0312	25.3721	**28.4178**
	SSIM	0.1858	0.3271	0.3882	0.3855	0.4857	0.5137	0.6449	0.7214	0.7926	**0.8802**
Mean	PSNR	15.3190	27.555	27.8438	28.1197	28.6098	29.2462	22.1526	24.0222	28.2102	**29.6265**
	SSIM	0.1198	0.2204	0.2537	0.2487	0.2966	0.3384	0.7757	0.7975	0.8540	**0.90165**

**Note:**

The best result in each row is shown in bold.

**Table 4:** The results of the proposed method based on PSNR (first rows) and SSIM (second rows) criteria on Zernike simulated datasets.

Sequence	Criteria	Proposed
Car-front	PSNR	23.0173
	SSIM	0.9208
Road	PSNR	31.7254
	SSIM	0.9167
Mean	PSNR	27.3714
	SSIM	0.9188

**Figure 3:**
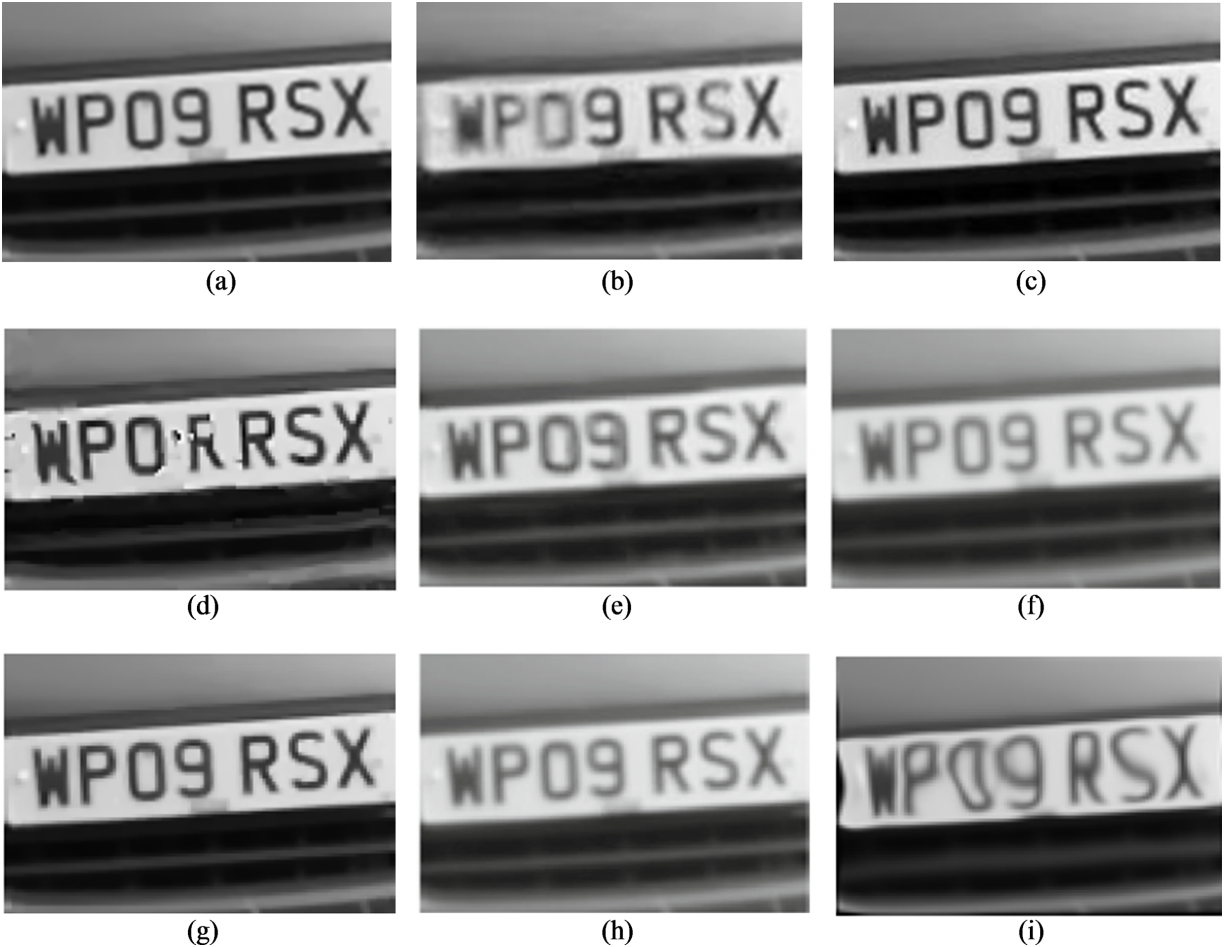
(A) The reference image of the car-front sequence (B) one observed image, and the qualitative results of (C) the proposed method, (D) NDL, (E) SGL, (F) centroid, (G) RPCA, (H) JSUB, and (I) two-stage, on this sequence. Photo credit: Pui Anantrasirichai.

**Figure 4:**
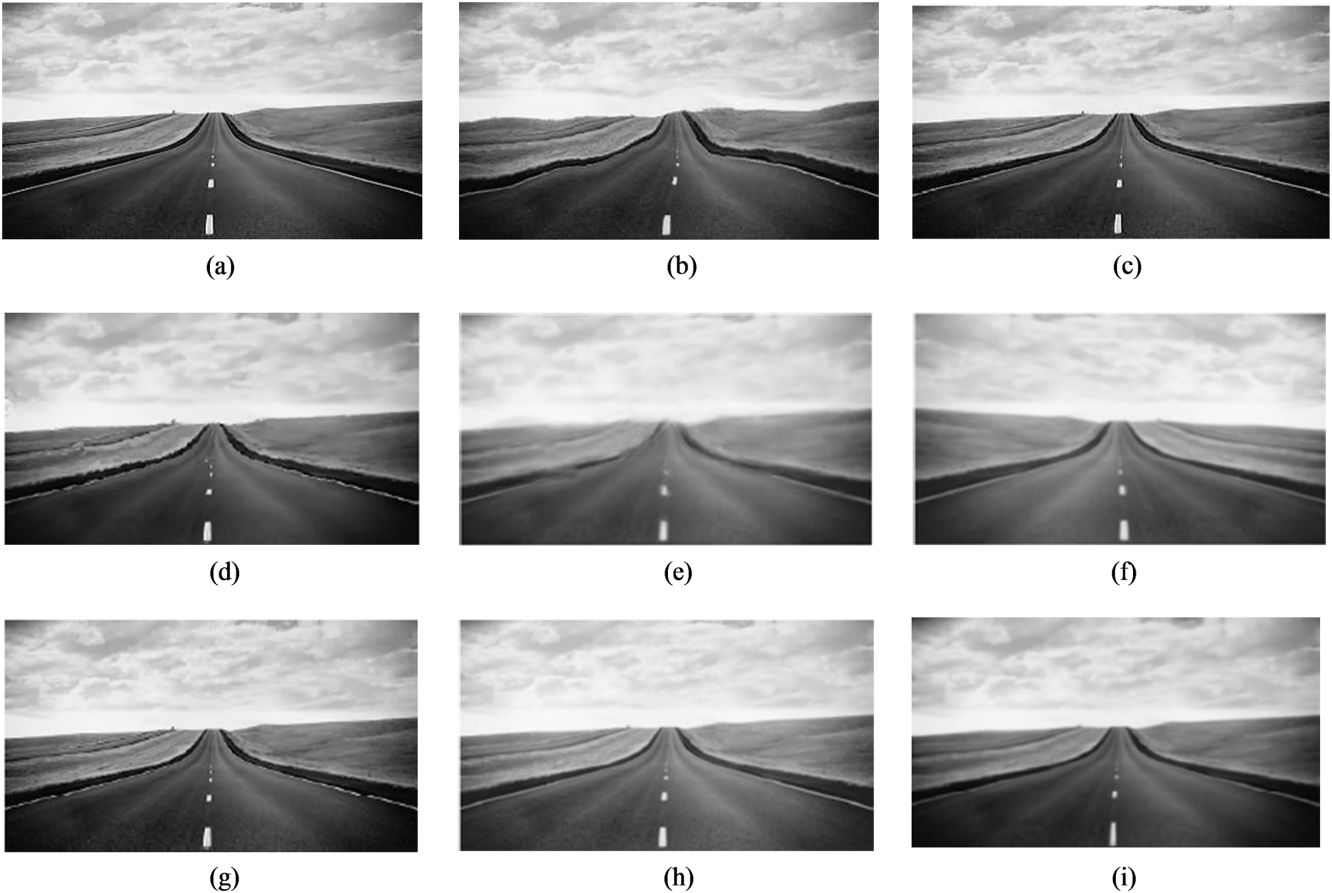
(A) The reference image of the road sequence, (B) one observed image, and the qualitative results of (C) the proposed method, (D) NDL, (E) SGL, (F) centroid, (G) RPCA, (H) JSUB, and (I) two-stage, on this sequence.

**Figure 5:**
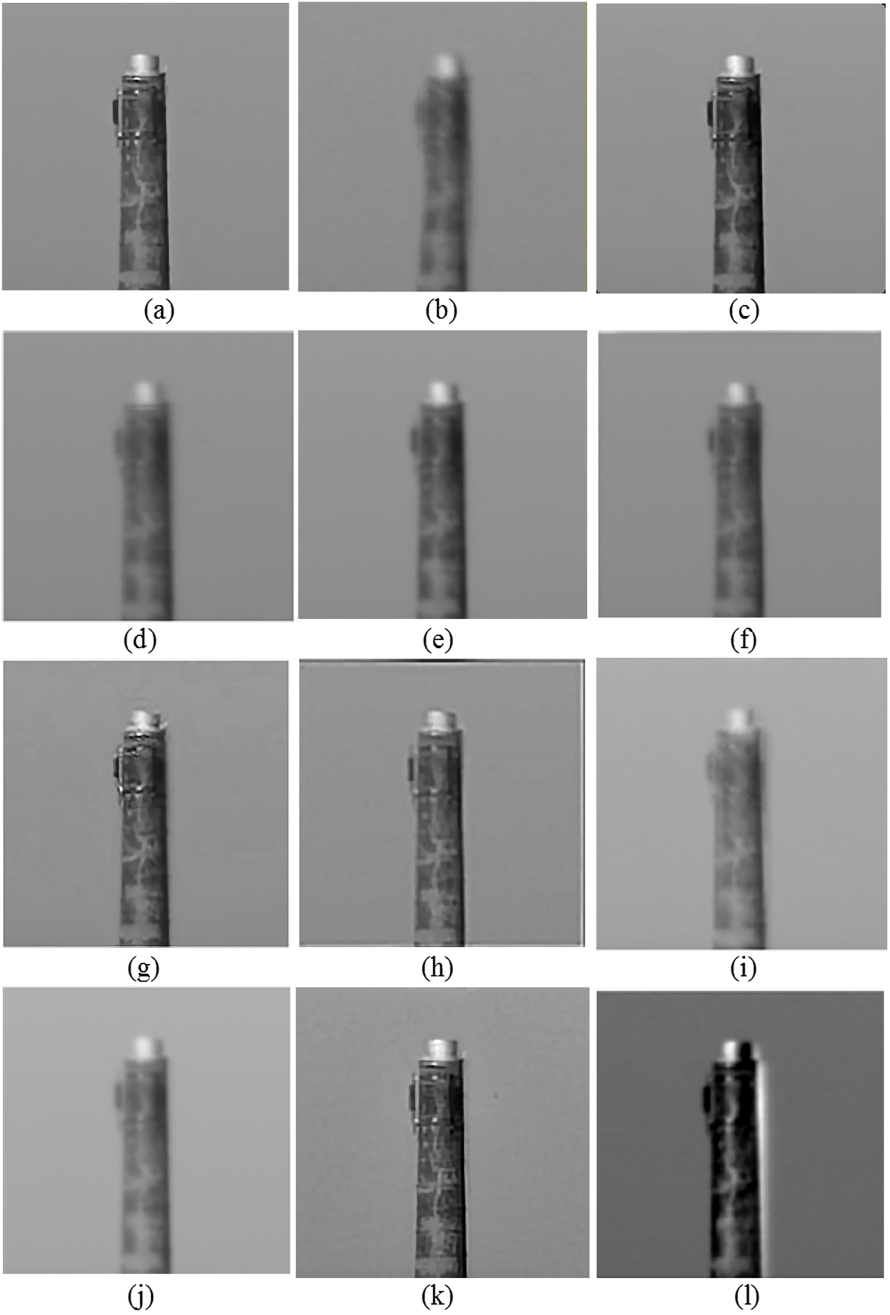
(A) The reference image of the chimney sequence, (B) one observed image, and the qualitative results of (C) the proposed method, (D) lucky region, (E) two-stage, (F) BNLTV, (G) NDL, (H) HVDKR, (I) SGL, (J) centroid, (K) UCSPF, and (L) PCA-based, on this sequence. Photo credit: Chiman Kwan.

**Figure 6:**
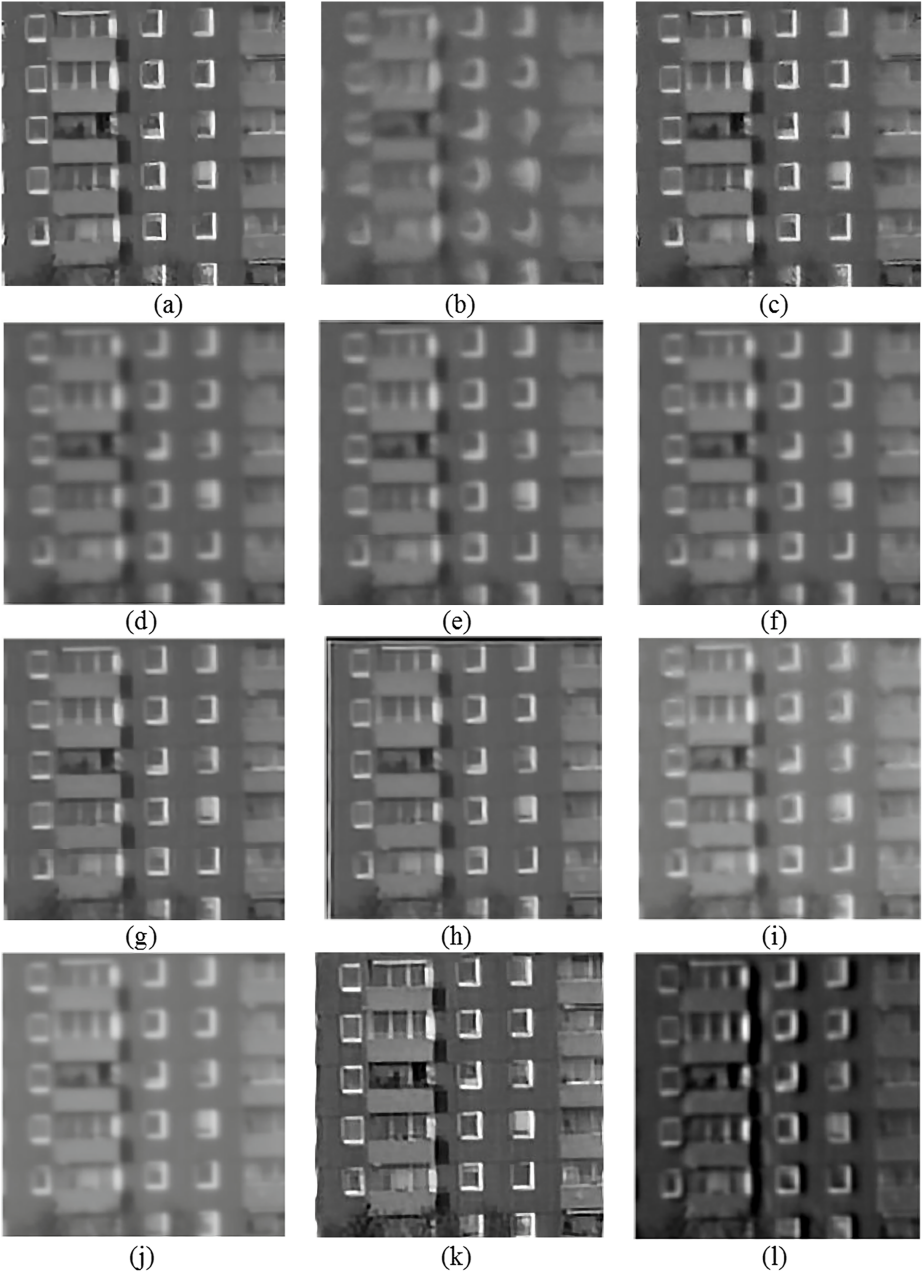
(A) The reference image of the building sequence, (B) one observed image, and the qualitative results of (C) the proposed method, (D) lucky region, (E) two-stage, (F) BNLTV, (G) NDL, (H) HVDKR, (I) SGL, (J) centroid, (K) UCSPF, and (L) PCA-based, on this sequence. Photo credit: Chiman Kwan.

Figures 3–6 provide visual representations of the restored images obtained from the proposed method and the results of the previous methods. It is evident from these images that the Centroid, RPCA, JSUB, Two-Stage, NDL, HVDKR, and UCSPF methods yield higher visual quality in terms of the final restored image compared to the other methods.

The Centroid method exhibits a restored image with lower quality compared to other methods. Despite the restoration of the geometric structure, the resulting image appears blurry and overly brightened due to the effects of temporal averaging.

The RPCA method, lacking a mechanism to effectively remove distortion, primarily focuses on selecting a subset of turbulent images with minimal distortion and maximal sharpness during the subsampling stage. As a result, the restored image obtained using the RPCA method continues to exhibit some degree of distortion. Nonetheless, due to its emphasis on the selection of a subset of sharp images and using the appropriate energy function to merge these subsampled images, coupled with the application of the blind deconvolution method, the restored image has little blur.

The JSUB method closely resembles the RPCA method except that its energy function uses TV regularization, the ${L_2}$ norm, and adaptive Gaussian noise for joint subsampling. This method also lacks a blind deconvolution step, resulting in a mildly distorted but blurred image.

The Two-Stage method’s deficiency caused by from the generation of an unsuitable reference image through the averaging process, leading to errors during the registration stage and ultimately resulting in a blurred final image.

Similar to the Two-Stage method, the NDL method also suffers from the impact of the erroneous reference image generated through the averaging process. This issue substantially affects the performance of the registration stage, and even though the method employs the blind deconvolution technique, the resulting image lacks clarity in terms of capturing image details. HVDKR uses the RPCA method to generate a reference image with reduced blur and distortion. This approach then further enhances the reference image using a variational model. Employing deformation-guided fusion the method not only models and mitigates the impact of the image but also effectively reduces blurriness in the final restored image. But since it is a step-by-step process, the interaction between the two factors of blur and distortion has not been taken into account. As a result, while the output images may exhibit relatively improved sharpness, the overall restored image tends to retain some level of blurriness and distortion, and also the fine details in the image are not well-defined.

In the UCSPF method, by separating the phase and magnitude components in the frequency domain, structural information and brightness intensity are separated to process the two distortion and blur factors separately. Through multiscale and multidimensional operations applied to these distinct components, the UCSPF method effectively produces an output image characterized by reduced levels of distortion and blur. Moreover, this approach facilitates the preservation of sufficient image details. Nonetheless, the processing conducted in the frequency domain lacks access to spatial information. For this reason, frequency-domain processing, although well-restored in detail in high-energy areas, has caused fluctuations in the brightness intensity in smooth regions. Despite the visually satisfactory quality of the final images, it is noteworthy that the computed PSNR and SSIM values are lower than expected.

**Figure 7:**
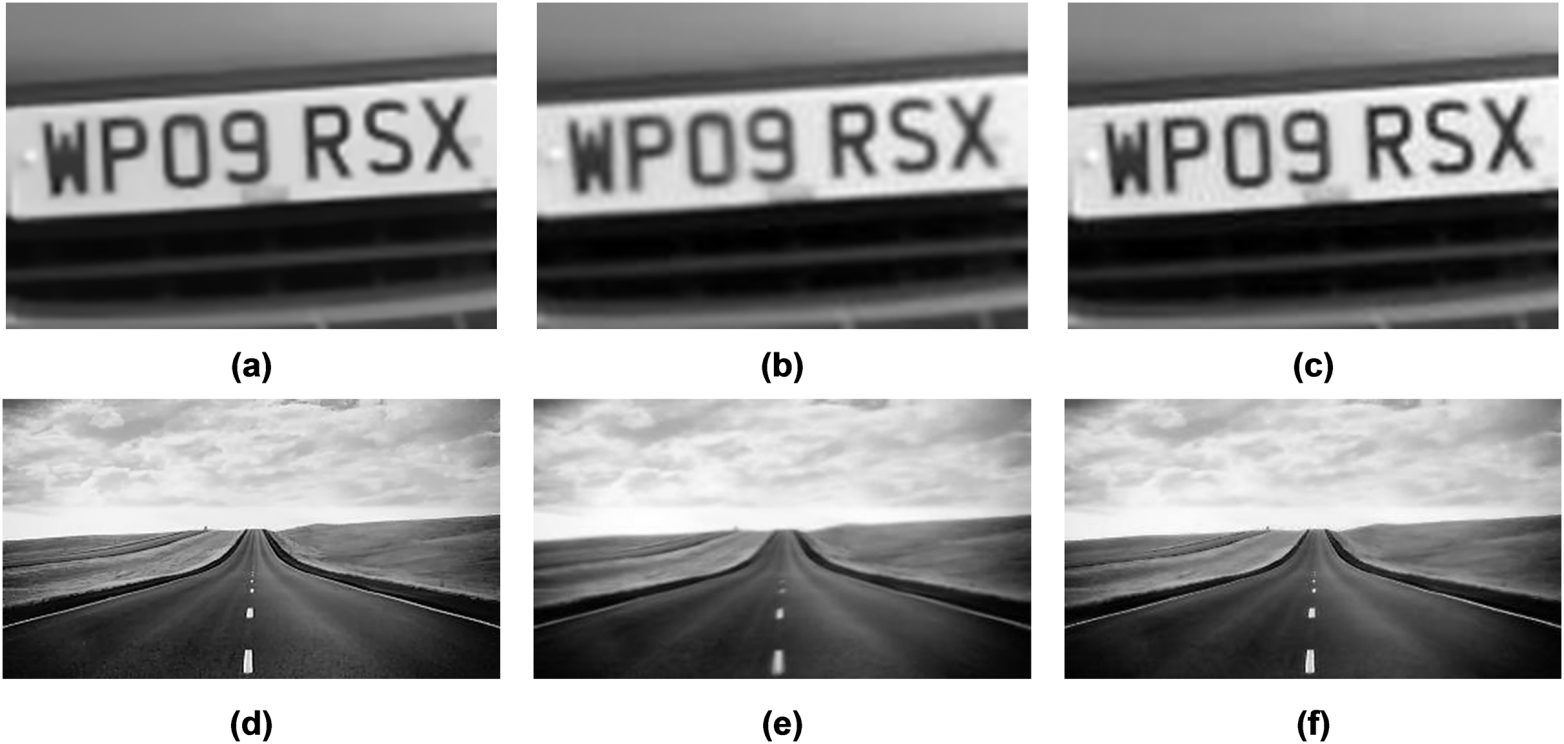
(A) The reference image of the car-front sequence (B) one Zernike simulated image, and (C) the qualitative results of the proposed method, (D) the reference image of the road sequence (E) one Zernike simulated image (F) the qualitative results of the proposed method. Photo credit: Pui Anantrasirichai.

As evident in Figs. 3–6, the resulting images from the proposed method exhibit sharper edges and finer details in comparison to those generated by other methods. The smooth regions within the ground truth images are returned as smoothly in the output images. The distortion is removed as much as possible, but the brightness of the output image in the Chimney sequence produced by the proposed method appears slightly darker compared to the corresponding ground truth image. Figure 7 presents the restored images generated by the proposed method on the Zernike turbulence dataset. A visual comparison of both Gaussian and Zernike turbulence images reveals that blurring is more obvious than distortion in the Zernike images. While the proposed method effectively restores Zernike turbulence images with notable accuracy, as evident in Fig. 7F, it is worth noting that the details of the asphalt road are not fully restored to their original quality.

Indeed, conventional images typically exhibit characteristics of low-frequency signals, rendering them low rank. Distortion, characterized by sudden change in the signal changes, introduces high-frequency components that elevate the signal’s rank.

The time-varying blur in an image sequence from a single scene results in images that are distinct from one another. Consequently, the overall diversity of the image sequence increases. This phenomenon elevates their temporal rank. Turbulence images, caused by both distortion and spatiotemporal varying blur, possess high ranks. Consequently, by generating a low-rank matrix, it becomes possible to obtain an image that encompasses all high-rank images derived from it. This image aims to closely resemble the reference (turbulence-free) image. Consequently, the creation of a low-rank matrix can be used to eliminate turbulence. Accordingly, the LRMF method was adopted for this particular objective. Through the iterative integration of these two techniques within the LRMF method, a comprehensive framework has been established. This framework is capable of generating turbulence-free images while minimizing the presence of turbulence. The performance of the proposed method was assessed in terms of the PSNR and SSIM criteria using the t-test statistical method. To accomplish this, the maximum values obtained from the previous methods were compared with those of the proposed method. The outcome of the PSNR-based t-test indicated a 50% similarity, signifying that the proposed method’s accuracy is on par with the best outcomes of the previous methods. Conversely, the t-test conducted based on the SSIM yielded a result of 95%, underscoring the superior accuracy of the proposed method in comparison to the preceding previous methods. Since the PSNR criterion is based on MSE, even slight changes in the image’s brightness can lead to a decrease in the numerical values of this criterion. Therefore, the relative darkening observed in the results of the proposed method might contribute to the decrease in the numerical values of this criterion. On the other hand, SSIM is a perception-based criterion that, like the human visual system, considers structural, luminance, and contrast similarities between the two images. Therefore, higher numerical values of this criterion in the results of the proposed method indicate a high perceptual similarity between the resultant images and the reference images. Further detailed evaluation of the proposed method is provided in Appendix D.

## Conclusion

In this article, a new method is proposed to remove turbulence effects in a sequence of images that simultaneously eliminates distortion and spatiotemporal varying blur. The approach involves modeling blur using a mixture of Gaussian (MOG) noise representation, while distortion is modeled as local geometric transformation matrices. Given that turbulence introduces high-rank characteristics to input images, we employ the low-rank matrix factorization (LRMF) technique to generate low-rank images with minimal turbulence. To address this, we introduce a novel framework that integrates two models, namely MOG noise and transformation matrices, into the LRMF method to effectively eliminate turbulence. Furthermore, accurate MOG parameter estimation and transformation matrix estimation are achievable for images with similar turbulence characteristics, we have leveraged the RANSAC method to identify and exclude images with distinct turbulence models. Our evaluation, conducted on both real and simulated datasets, consistently demonstrates that the proposed method outperforms its predecessors. This method does have its limitations, including relatively slower processing speed compared to certain previous methods, as well as instances where the resulting images may appear darker than the reference image. While the proposed method demonstrates a degree of robustness to variations in its free parameters, the experiments conducted across diverse datasets indicate that the values suggested in this article generally lead to satisfactory accuracy in the output. However, if these free parameters were fine-tuned based on the images used, better results could be achieved.

## Future work

Indeed, the proposed method encompasses numerous free parameters that require expert regulation. While these parameters might not significantly impact the method’s performance and processing speed, the intention behind offering adjustable parameters is to allow practitioners to tailor them according to specific image conditions. This adaptability ensures that other developers can readily apply the method to their own applications with ease. Enhancing the speed of the proposed method is feasible by incorporating a frequency-domain distortion modeling approach. This strategy involves introducing an appropriate term during the noise removal process, thereby generating an image that more closely aligns with the ground truth in terms of brightness. This frequency-domain distortion modeling technique holds potential for improving both the computational efficiency and the quality of the final output image. One of the authors’ future plans involves extending this method to address turbulence removal in dynamic environments that include moving objects. This expansion would center around refining the estimation of the transformation matrix model to account for changes caused by the presence of moving objects within the scene.

## Supplemental Information

10.7717/peerj-cs.1713/supp-1 Supplemental Information 1 Appendix.

10.7717/peerj-cs.1713/supp-2 Supplemental Information 2 Source Code and Dataset.

10.7717/peerj-cs.1713/supp-3 Supplemental Information 3 (A) The reference image of the Water tower sequence, (B) one observed image, and the qualitative results of (C) the proposed method, (D) Centroid, (E) NDL, (F) RPCA, (G) SGL, and (H) Two-Stage, on this sequence.

10.7717/peerj-cs.1713/supp-4 Supplemental Information 4 (A) The reference image of the Moon surface sequence, (B) one observed image, and the qualitative results of (C) the proposed method, and (D) NDL, on this sequence.

10.7717/peerj-cs.1713/supp-5 Supplemental Information 5 Demonstration of different turbulence impact on Desert sequence. (A) Desert sequence reference image, (B) a weak turbulence sample, and (C) a severe turbulence sample

10.7717/peerj-cs.1713/supp-6 Supplemental Information 6 The effect of the turbulence rate on the accuracy of the proposed method (PSNR). Sequences with the smaller number represent the images with weaker turbulence.

10.7717/peerj-cs.1713/supp-7 Supplemental Information 7 The effect of the alpha parameter on the accuracy of the proposed method.

10.7717/peerj-cs.1713/supp-8 Supplemental Information 8 The effect of putting aside the (A) distortion factor, (B) blurring factor, and (C) RANSAC on the performance of the proposed method on the Car-front sequence.

10.7717/peerj-cs.1713/supp-9 Supplemental Information 9 List of symbols and variables.

10.7717/peerj-cs.1713/supp-10 Supplemental Information 10 Zernike Simulation Parameters.

10.7717/peerj-cs.1713/supp-11 Supplemental Information 11 Detailed information about the datasets employed in experiments.

10.7717/peerj-cs.1713/supp-12 Supplemental Information 12 The results of the proposed method on the Moon-surface and Water-tower sequences.

10.7717/peerj-cs.1713/supp-13 Supplemental Information 13 The comparison of the results of the proposed method by putting aside different components on the Car-front sequence based on the SSIM and PSNR criteria.

## Additional Information and Declarations

### Data Availability

The following information was supplied regarding data availability: The source code, datasets and brief instruction for executing the codes are available in the Supplemental File.
