# Supplementary material for: A new approach for atmospheric turbulence removal using low-rank matrix factorization
Source: PeerJ Comput Sci. 2024 Jan 31;10:e1713. doi: 10.7717/peerj-cs.1713 (PMC10909186; doi:10.7717/peerj-cs.1713)
Supplement: Supplemental Information 1 [file peerj-cs-10-1713-s001.docx]

**Appendices**

**Appendix A: Calculating the similarity of two MOG distributions**

The KL divergence method can be used to calculate the similarity between two Mixture of Gaussian (MOG) distributions. Let $p\left( x \right)=N\left( \mu_{1},\Sigma_{1} \right)$, and $q\left( x \right)=N\left( \mu_{2},\Sigma_{2} \right);$then, the KL divergence between the two distributions $p\left( x \right)$and$q\left( x \right)$is obtained from Eq. (S1):

$KL\left( p,q \right)=\sum_{x} p\left( x \right)log(p\left( x) \right)-\sum_{x} p\left( x \right)log(q\left( x \right))$ (S1)

Now, let $p\left( x \right),$and$q\left( x \right)$ be the MOG distributions, with$p\left( x \right)=\sum_{i=1}^{n} \alpha_{i}N\left( \mu_{1,i},\Sigma_{1,i} \right) and q\left( x \right)=\sum_{j=1}^{m} \beta_{j}N\left( \mu_{2,j},\Sigma_{2,j} \right)$. As stated in Goldberger et al. (2003), to calculate the KL divergence between the two distributions $p$ and$q,$it is necessary to find the closest Gaussian model in $q\left( x \right)$ for each Gaussian model of $p\left( x \right)$. To this purpose, the matching function $\pi:\left\{ 1,2,\ldots,n \right\}\to\{1,2,\ldots,m\}$ is presented as Eq. (S2) between the models $p$and$q$:

$\pi\left( i \right)=\underset{j}{\mathrm{argmin}} (\frac{1}{2}(\log\frac{\left| \Sigma_{2,j} \right|}{\left| \Sigma_{1,i} \right|}+Tr\left( \Sigma_{2,j}^{-1}\Sigma_{1,i} \right)+{{(\mu}_{1,i}-\mu_{2,j})}^{T}\Sigma_{2,j}^{-1}{(\mu}_{1,i}-\mu_{2,j}))-\log\beta_{j})$ (S2)

Where $Tr$represents the trace of the matrix. Consequently, the KL divergence between the two MOG distributions *p* and *q* can be expressed as follows:

$KL\left( p,q \right)= \sum_{i=1}^{n} \alpha_{i}(KL\left( p_{i},q_{\pi\left( i \right)} \right)+\log\frac{\alpha_{i}}{\beta_{\pi(i)}})$ (S3)

**Appendix B: Dataset details**

generate the image sequence for the simulated dataset, the original images within these sets are initially subdivided into overlapping patches, each with a size of 65x65. The total count of these patches is determined by$\frac{Width*Height}{D_{i}}$​, with the parameter $D_{i}$set to a value of 250. Subsequently, a deformation vector set is created for each of these patches, employing a Gaussian distribution. Each set of vectors is smoothed and multiplied by a random number (known as the distortion coefficient). Following this, each individual patch is warped using its final corresponding vector. Lastly, each image sequence is blurred by different Gaussian noise. The distortion coefficient for severe distortion falls within the range of [1.0, 1.5], while for moderate distortion, it lies within the range of [0.2, 0.3]. The road image sequence comprises 70 images with severe distortion and 30 images with moderate distortion. Conversely, the Car-front image sequence exclusively displays images with moderate distortion.

Chimitt and Chan (2020) introduced a propagation-free method for simulating turbulence images. Drawing on the findings of Noll (1976), they focused on utilizing the *2nd* and *3rd* Zernike coefficients as deformation vectors, while designating other high-order Zernike coefficients for the purpose of blurring. To generate the image sequence through this approach, the initial images are divided into blocks of size 16×16. Subsequently, spatially varying blur is applied to these blocks, with the blur calculated based on high-order Zernike coefficients. Ultimately, the entire image is warped employing motion vectors, which are determined using the *2nd* and *3rd* Zernike coefficients. A total of 100 turbulence images were produced utilizing this method. The detailed parameters used in the proposed method, extracted from the reference paper, are presented in Table S2 (Chimitt et al., 2020). Real image sequences were captured in a real turbulence mode that includes both factors, spatiotemporal varying blur and distortion. The Building and Chimney sequences were captured from a building vent that exhausted hot air. The Moon-surface sequence was captured using a ground-based telescope. The Water-tower sequence was shot at a distance of 2.4 kilometers (1.5 miles) away from the water tower, which was situated atop a non-uniform terrain. In all the real sequences that were captured under controlled conditions, spatiotemporal varying blur is dominant over distortion. The specifics of the datasets are presented in Table S3. These datasets are accessible online at <https://github.com/jafaeimahdi/Atmospheric-Turbulence-DATA>.

**Appendix D: PSNR and SSIM criteria**

The PSNR criterion employs the root mean square error between two images. Eq. (S4) shows this criterion:

$MSE= \frac{1}{n_{1}n_{2}}\sum_{i=0}^{n_{1}-1} \sum_{j=0}^{n_{2}-1} \left[ x_{1}\left( i,j \right)-x_{2}\left( i,j \right) \right]^{2}$ (S4)

$PSNR=20 {log}_{10} (\frac{MAX}{\sqrt{MSE}})$ (S5)

Where $n_{1} \text{and} n_{2}$​ represent the pixel counts in the first and second images, respectively. For images with 8-bit pixel values, $MAX$ is equal to 255. The SSIM formula is presented in Eq. (S9):

$L\left( x_{1},x_{2} \right)= \frac{2\mu_{x_{1}}\mu_{x_{2}}+c_{1}}{\mu_{x_{1}}^{2}+\mu_{x_{2}}^{2}+c_{1}}$ (S6)

$C\left( x_{1},x_{2} \right)= \frac{2\sigma_{x_{1}}\sigma_{x_{2}}+c_{2}}{\sigma_{x_{1}}^{2}+\sigma_{x_{2}}^{2}+c_{2}}$ (S7)

$S\left( x_{1},x_{2} \right)=\frac{\sigma_{x_{1}x_{2}}+ c_{3}}{\sigma_{x_{1}}\sigma_{x_{2}}+c_{3}}$ (S8)

Where $\mu_{x_{1}},\mu_{x_{2}},\sigma_{x_{1}},\sigma_{x_{2}}, \text{and} \sigma_{x_{1}x_{2}}$ are, respectively, the mean of pixel values in the first image, the mean of pixel values in the second image, the variance of pixel values in the first image, the variance of pixel values in the second image, and the covariance of pixel values between the first and second images; $c_{1},c_{2}, \text{and} c_{3}$ are variables introduced to prevent division by zero. If $c_{3}=\frac{c_{2}}{2}$ , then, SSIM becomes a weighted combination of *S*, *C*, and *L*:

$SSIM\left( x_{1},x_{2} \right)= \left[ {L\left( x_{1},x_{2} \right)}^{\alpha}{C\left( x_{1},x_{2} \right)}^{\beta}{S\left( x_{1},x_{2} \right)}^{\gamma} \right]$ (S9)

If $\alpha=\beta=\gamma=1$, we have

$SSIM\left( x_{1},x_{2} \right)=\frac{(2\mu_{x_{1}}\mu_{x_{2}}+c_{1})(2\sigma_{x_{1}x_{2}}+ c_{2})}{(\mu_{x_{1}}^{2}+\mu_{x_{2}}^{2}+c_{1})(\sigma_{x_{1}}^{2}+\sigma_{x_{2}}^{2}+c_{2})}$ (S10)

**Appendix D: Comprehensive evaluation of the proposed method**

**Water-tower and Moon-surface results**

As detailed in the main text of the manuscript, this section exclusively presents the outcomes derived from the proposed method for the Water-tower and Moon-surface datasets. The qualitative comparison between the results of the proposed method, the original images, and the turbulence images can be observed in Figs S1 and S2. Moreover, the results of the proposed method on the sequences of Moon-surface and Water-tower images are shown in Table S4.

**Effect of Image Complexity**

An investigation was conducted into the impact of image complexity on the performance of the proposed method. To achieve this, the correlation between SSIM and the final image energy, which is equivalent to the image gradient, was computed across all tested image sequences. The outcome of this calculation yielded a value of 0.4433.

Hence, it can be deduced that higher image energy correlates with improved performance of the proposed method. Conversely, in instances where the image has larger smooth areas, characterized by low energy, the accuracy of the proposed method diminishes. These conclusions are also supported by the information presented in Tables 2 and 3 within the main text of the manuscript, as well as Tables S4. The reason is that because distortion is modeled based on local transformation, in smooth areas where there is less detail, the uncertainty is increased and thus the accuracy of the distortion estimation is reduced.

**Effect of turbulence rate**

To examine the impact of turbulence rate on the efficacy of the proposed method, a sequence of turbulent images with different turbulence rates was generated using a desert image as a source (Lau et al., 2019). The selection of the desert image, with dimensions of 240×300 pixels, is well-suited for this analysis due to its combination of smooth and detailed areas and also, like pictures taken from man-made structures, the image encompasses a sufficient number of edges. In order to generate a sequence of turbulent images with all the lower to higher values of distortion and blur, two parameters were defined: the blur parameter *σ*, with values ranging from 0.01 to 2, and the distortion coefficient parameter with values ranging from 0.1 to 2. Consequently, a total of 100 sets of turbulent images, each consisting of 100 images, were generated in this sequence, and all the images within each set exhibit an identical level of turbulence. The reference image for the desert sequence, along with two examples of weakly and severely turbulent images can be observed in Fig. S3. Additionally, Figure S4 illustrates the graphical representation of the impact of turbulence rate on the performance of the proposed method, as evaluated through the PSNR criteria. As depicted in the diagram, the proposed framework exhibits suboptimal performance in the case of weak turbulence. This outcome can be attributed to the fact that weak turbulence yields images that are relatively alike. Consequently, during the process of blur removal and the estimation of the *U* and *V* low-rank matrices, the turbulence is also interpreted as an intrinsic element of the final image. This phenomenon subsequently diminishes the overall accuracy of the system. Through the augmentation of turbulence rate and the amplification of turbulence's impact on the image, the proposed method discerns these variations and more effectively mitigates the turbulence's influence. As a result, the quality of the output image is improved, and the algorithm's accuracy is enhanced. As indicated by the diagram, when the turbulence rate is higher, the system's performance diminishes with a slight slope due to severe distortions within the image. During such instances, the process of eliminating distortion cannot properly estimate local transformation matrices, resulting in a reduction in the performance of the proposed method. Consequently, it can be deduced that the proposed method achieves higher performance for intermediate turbulence. Nevertheless, as depicted in the diagram, there exists a slight difference (approximately 0.3) between the lowest and highest performances of the proposed method as evaluated using the PSNR criteria.

**Investigating the effects of free parameters**

The most important parameter that affects the performance of the proposed method is the $\alpha$parameter, which is highly dependent on the input image conditions. The selection of an appropriate value for this parameter, which generally produces acceptable results for all datasets, is of particular importance; it has a direct impact on how distortion is modeled. To investigate the impact of this parameter on the accuracy of the proposed method, the whole set of experimental datasets with different values of $\alpha\left( 0<\alpha<1 \right)$is investigated, and the average result is shown in Fig. S5.

As Fig. S5 shows, the close-to-one values ​​of $\alpha$make it difficult to achieve the $\alpha$ percent of image entropy, so most of the windows are expanded to the maximum size of $W_{max},$ which means they are considered outliers. Due to the substantial presence of outlier windows, the algorithm encounters challenges in accurately modeling distortion. Through the reduction of $\alpha$, the intended entropy value per window is lowered. This adjustment effectively maintains the size of the majority of windows within the range of $W_{min}$​ and $W_{max},$​, consequently minimizing the occurrence of outlier windows.

At the peak point, the window size attains a relatively optimized state. It achieves a balance, neither being too small to effectively model local transformations nor excessively large that it goes out of locality and includes multiple transformations. Further reduction in the $\alpha$ value leads to a diminishment in the size of windows necessitating the achievement of this entropy, and due to the distance from the semi-optimal window size and the lack of proper modeling of the transformations, the accuracy of the algorithm decreases until eventually $\alpha$ becomes so small that all the selected windows will have the smallest allowable window size ($W_{min}$); the algorithm's accuracy remains constant from that point since, given the window size's absence of change.

**Effects of Proposed Method Components**

The proposed method comprises three primary components: RANSAC, spatiotemporal varying blur estimation, and distortion estimation. In this section, the effects of modeling the distortion and blurring factors and the RANSAC method on the performance of the proposed algorithm are investigated separately. For this purpose, in the proposed method, the noise removal process, which includes the estimation of the parameters $\Pi\text{and} \Sigma$, the process of calculating the transformation matrices, and the RANSAC procedure were put aside, separately. It's important to note that excluding the $\Pi\text{and} \Sigma$ parameters is equivalent to setting the weights of the Weighted Alternating Least Squares (WALS) method to one. To assess the proposed method's performance in these three scenarios, the Car-front sequence was employed as the evaluation dataset. The obtained results are illustrated in Figure S6.

As demonstrated, when the distortion removal process is omitted (Fig. S6.a), noticeable letter distortions become apparent. On the other hand, omitting the blurring elimination process (Fig. S6.b) leads to a substantial reduction in letter distortions, although the image appears more blurred compared to Fig. S6.a. Notably, when the RANSAC procedure is omitted, both distortion and blurring effects are mitigated. Nevertheless, when compared to the results of the proposed method, edges appear smoother and slight letter distortions are still discernible.

Table S5 also shows the result of these three modes according to the SSIM and PSNR criteria. While the simultaneous integration of noise removal and transformation matrix computation exhibited robust performance for the proposed method, evident from the elevated SSIM and PSNR values, excluding the distortion removal process led to a further decline in accuracy of the proposed method; thus, modeling distortion (computing the transformation matrix) holds greater efficacy in enhancing the proposed method's performance compared to modeling blurring. Notably, while the simultaneous execution of both distortion and blurring processes yielded favorable outcomes, each process individually failed to achieve satisfactory results. Through a comparison of the SSIM and PSNR outcomes between the proposed method and the outcomes achieved by excluding the RANSAC procedure, it becomes evident that excluding images with turbulences different from other images can substantially contribute to enhancing the accuracy of the proposed method. Because of the limitation in MOG model numbers, if the blurring image sequence includes very different blurring models, the MOG cannot model blurring well. So, it is more accurate to estimate MOG parameters for images with similar blurring models than for images with different blurring models. Since MOG is employed in the distortion estimation too, the situation is the same for distortion estimation. It should be emphasized that in the proposed method, RANSAC wasn't utilized to choose either weak or severe turbulence images: it was used to select similar turbulence images. Thus, using the RANSAC method to select images with a similar turbulence model improves the performance of the turbulence removal process.

**Appendices References**

**Goldberger, J., Gordon, S. and Greenspan, H. 2003.** An Efficient Image Similarity Measure Based on Approximations of KL-Divergence Between Two Gaussian Mixtures. In ICCV (Vol. 3, pp. 487-493).

**Chimitt, N. and Chan, S.H., 2020.** Simulating anisoplanatic turbulence by sampling intermodal and spatially correlated Zernike coefficients. Optical Engineering, 59(8), pp.083101-083101.

**Noll, R.J.**, 1976. Zernike polynomials and atmospheric turbulence. JOsA, 66(3), pp.207-211.

**Lau, C.P., Lai, Y.H. and Lui, L.M. 2019.** Restoration of atmospheric turbulence-distorted images via RPCA and quasiconformal maps. Inverse Problems, 35(7), p.074002.
