# Supplementary figures and images for: A new approach for atmospheric turbulence removal using low-rank matrix factorization
Source: PeerJ Comput Sci. 2024 Jan 31;10:e1713. doi: 10.7717/peerj-cs.1713 (PMC10909186; doi:10.7717/peerj-cs.1713)

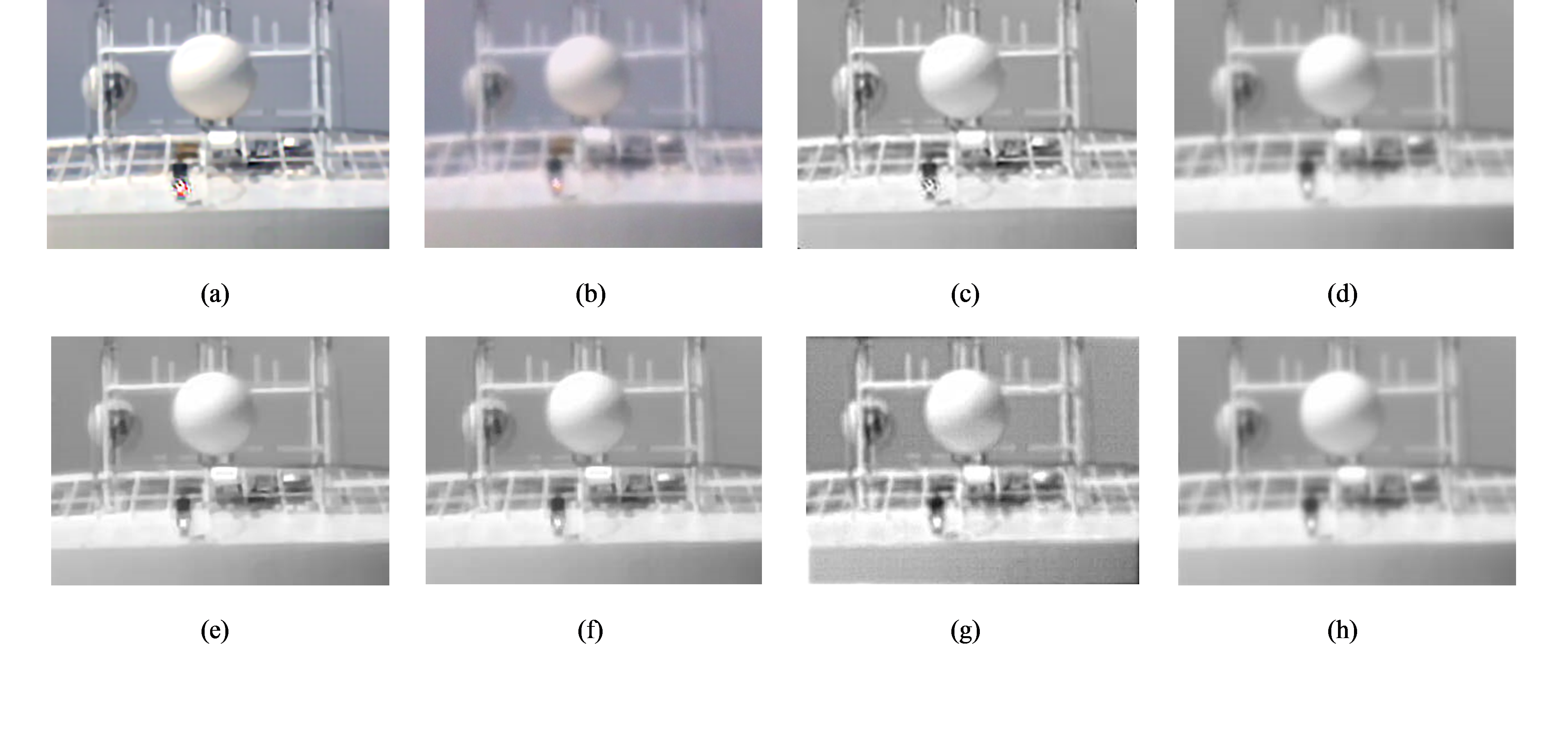

Supplement: Supplemental Information 3 [file peerj-cs-10-1713-s003.png]

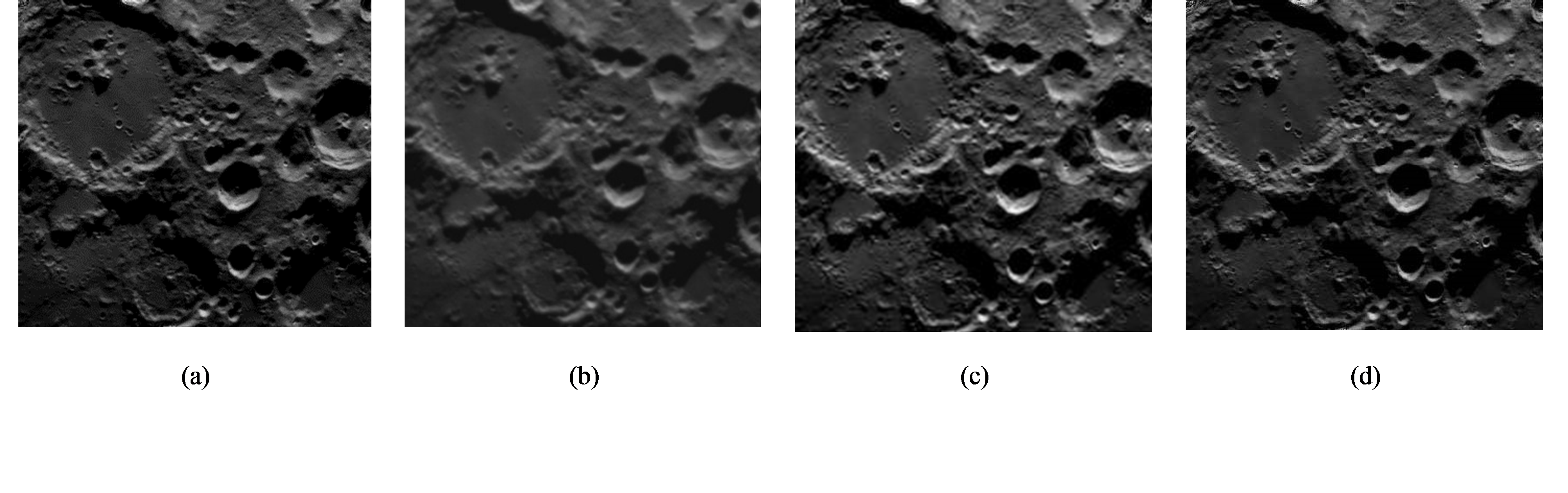

Supplement: Supplemental Information 4 [file peerj-cs-10-1713-s004.png]

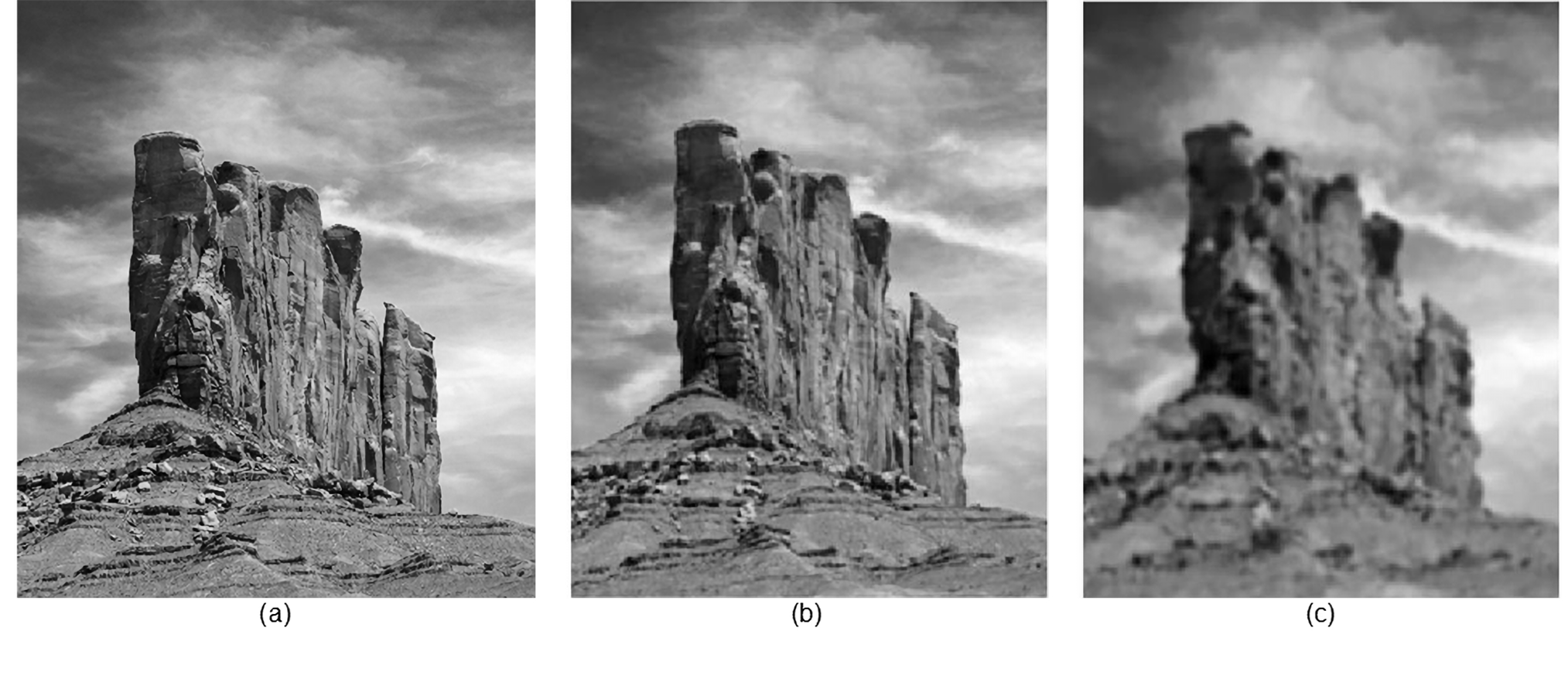

Supplement: Supplemental Information 5 — (A) Desert sequence reference image, (B) a weak turbulence sample, and (C) a severe turbulence sample [file peerj-cs-10-1713-s005.png]

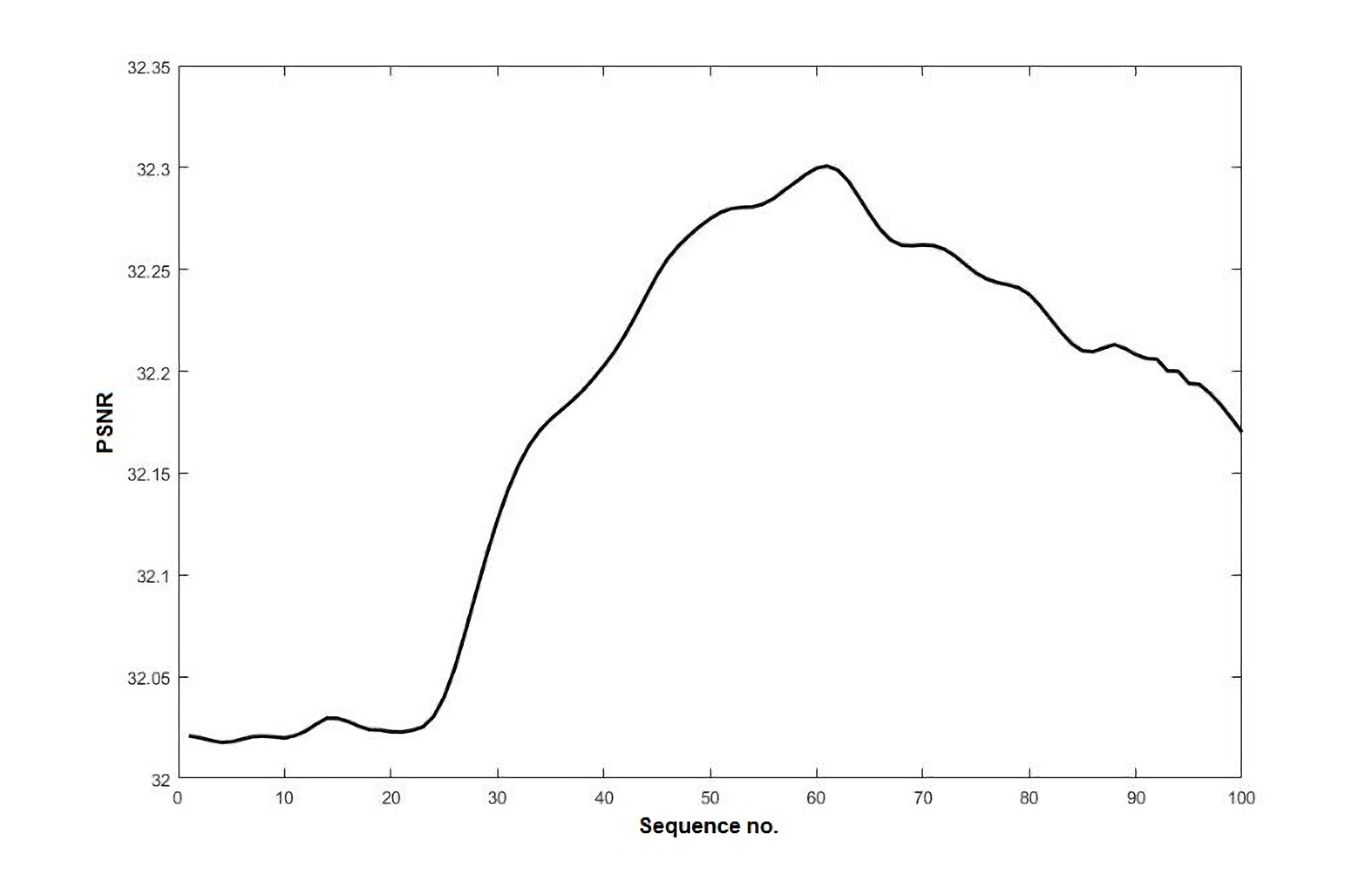

Supplement: Supplemental Information 6 — Sequences with the smaller number represent the images with weaker turbulence. [file peerj-cs-10-1713-s006.png]

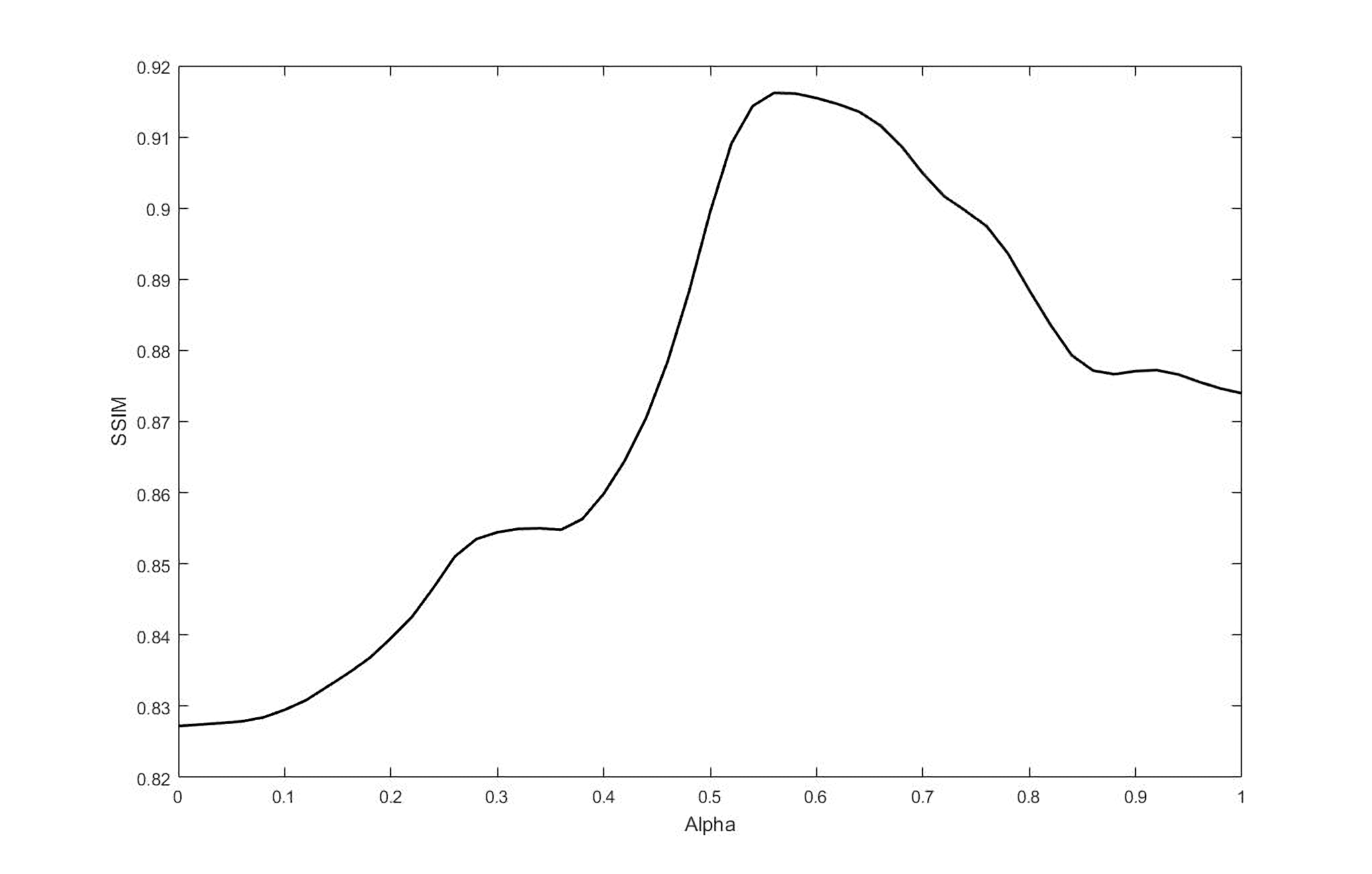

Supplement: Supplemental Information 7 [file peerj-cs-10-1713-s007.png]

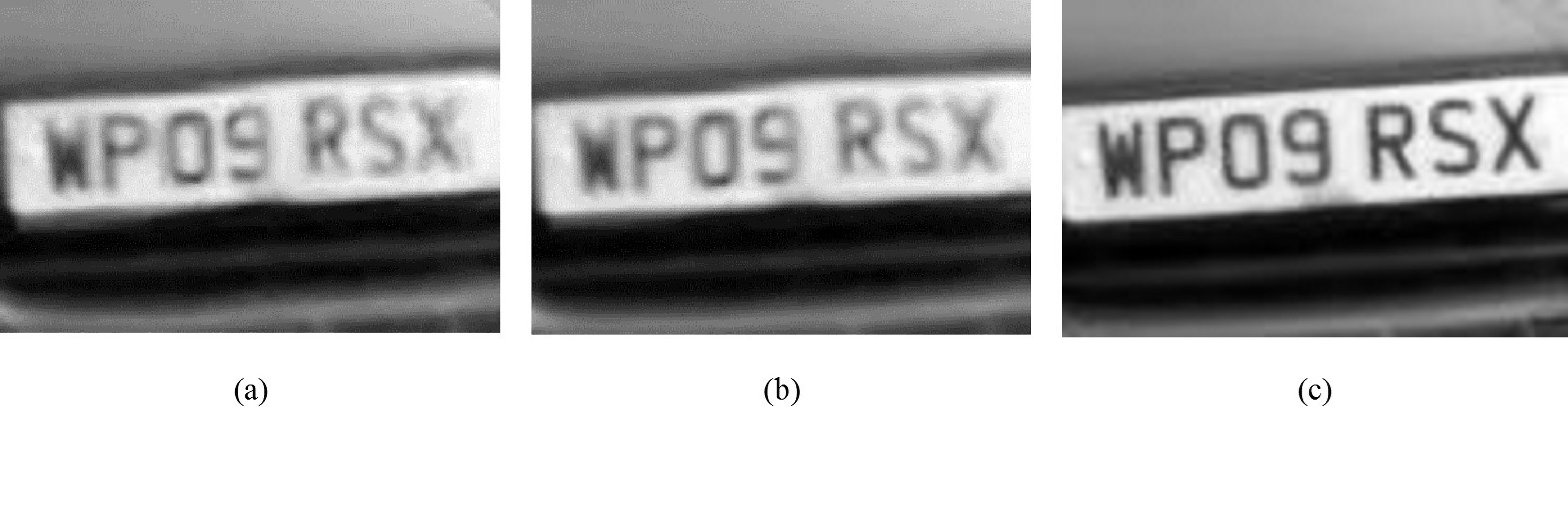

Supplement: Supplemental Information 8 [file peerj-cs-10-1713-s008.jpg]
