# Supplementary material for: A new approach for atmospheric turbulence removal using low-rank matrix factorization
Source: PeerJ Comput Sci. 2024 Jan 31;10:e1713. doi: 10.7717/peerj-cs.1713 (PMC10909186; doi:10.7717/peerj-cs.1713)
Supplement: Supplemental Information 9 [file peerj-cs-10-1713-s009.docx]

| **Table S1 List of symbols and variables.** | |
| --- | --- |
| **Parameter or symbol** | **Description** |
| $X$ | Sequence of turbulent images |
| $n$ | Number of images |
| $d$ | Dimension of each turbulent image |
| $U$ | Basis matrix |
| $V$ | Coefficient matrix |
| $u_{i}$ | $i^{th}$ row vector of *U* |
| $v_{j}$ | $j^{th}$ row vector of *V* |
| $\varepsilon_{ij}$ | Image noise per $j^{th}$ pixel of the $i^{th}$ turbulent image |
| $N \left( 0, \sigma_{k}^{2} \right)$ | Gaussian distribution with zero mean and $\sigma^{2}$ variance |
| $W$ | Matrix of non-negative weights |
| $Y$ | Turbulence-free images |
| $diag\left( w_{i} \right)$ | Diagonal matrix with elements $w_{i}$ |
| $I$ | Identity matrix |
| $\tau$ | Transformation matrix |
| $\circ$ | Transformation operator |
| $J_{i}$ | Jacobian matrix of $i^{th}$ image |
| $S_{\frac{1}{\mu}}$ | Soft thresholding operator |
| $\rho$ | Penalty value in ADMM algorithm |
| *P* | Step of the ADMM algorithm |
| $W_{min}$ | Minimum allowable size of window |
| $W_{max}$ | Maximum allowable size of window |
