# Supplementary material for: A new approach for atmospheric turbulence removal using low-rank matrix factorization
Source: PeerJ Comput Sci. 2024 Jan 31;10:e1713. doi: 10.7717/peerj-cs.1713 (PMC10909186; doi:10.7717/peerj-cs.1713)
Supplement: Supplemental Information 10 [file peerj-cs-10-1713-s010.docx]

**Table S2 Zernike Simulation Parameters.**

| **Parameter** | **Value** |
| --- | --- |
| Path length | L = 7km |
| Aperture Diameter | D = 0.2034m |
| Focal Length | d = 1.2m |
| Wavelength | λ= 525nm |
| Zernike Phase Size | 16 × 16 pixels |
| Nyquist spacing (object plane) | $\delta_{o}$ = 9:0344mm |
| Nyquist spacing (focal plane) | $\delta_{f}$ = 1:5488_m |
