# Supplementary material for: A new approach for atmospheric turbulence removal using low-rank matrix factorization
Source: PeerJ Comput Sci. 2024 Jan 31;10:e1713. doi: 10.7717/peerj-cs.1713 (PMC10909186; doi:10.7717/peerj-cs.1713)
Supplement: Supplemental Information 11 [file peerj-cs-10-1713-s011.docx]

**Table S3 Detailed information about the datasets employed in experiments.**

| Type | Name | Resolution | # images |
| --- | --- | --- | --- |
| Simulated | Road  Car-front | 350×196  120×80 | 100  100 |
| Real | Moon surface  Water tower  Chimney  Building | 410×380  300×220  240×240  240×240 | 80  80  100  100 |
