# Supplementary material for: A new approach for atmospheric turbulence removal using low-rank matrix factorization
Source: PeerJ Comput Sci. 2024 Jan 31;10:e1713. doi: 10.7717/peerj-cs.1713 (PMC10909186; doi:10.7717/peerj-cs.1713)
Supplement: Supplemental Information 12 [file peerj-cs-10-1713-s012.docx]

| **Table S4 The results of the proposed method based on PSNR (first rows) and SSIM (second rows) criteria on the Moon-surface and Water-tower sequences.** | | |
| --- | --- | --- |
| Proposed | Criteria | Sequence |
| **27.3678** | PSNR | Moon surface |
| **0.9257** | SSIM |  |
| **31.3197** | PSNR | Water tower |
| **0.9436** | SSIM |  |
| **29.3437** | PSNR | Mean |
| **0.9346** | SSIM |  |
