# Supplementary material for: A new approach for atmospheric turbulence removal using low-rank matrix factorization
Source: PeerJ Comput Sci. 2024 Jan 31;10:e1713. doi: 10.7717/peerj-cs.1713 (PMC10909186; doi:10.7717/peerj-cs.1713)
Supplement: Supplemental Information 13 [file peerj-cs-10-1713-s013.docx]

| **Table S5 The comparison of the results of the proposed method by putting aside different components on the Car-front sequence based on the SSIM and PSNR criteria.** | | | | |
| --- | --- | --- | --- | --- |
| Criteria | The proposed method without distortion detection | The proposed method without blur removal | The proposed method without RANSAC procedure | The proposed method |
| SSIM | 0.6919 | 0.7612 | 0.8207 | 0.9460 |
| PSNR | 17.74 | 18.66 | 20.67 | 25.1344 |
